# Reliability and accuracy of single-molecule FRET studies for characterization of structural dynamics and distances in proteins

**DOI:** 10.1038/s41592-023-01807-0

**Published:** 2023-03-27

**Authors:** Ganesh Agam, Christian Gebhardt, Milana Popara, Rebecca Mächtel, Julian Folz, Benjamin Ambrose, Neharika Chamachi, Sang Yoon Chung, Timothy D. Craggs, Marijn de Boer, Dina Grohmann, Taekjip Ha, Andreas Hartmann, Jelle Hendrix, Verena Hirschfeld, Christian G. Hübner, Thorsten Hugel, Dominik Kammerer, Hyun-Seo Kang, Achillefs N. Kapanidis, Georg Krainer, Kevin Kramm, Edward A. Lemke, Eitan Lerner, Emmanuel Margeat, Kirsten Martens, Jens Michaelis, Jaba Mitra, Gabriel G. Moya Muñoz, Robert B. Quast, Nicole C. Robb, Michael Sattler, Michael Schlierf, Jonathan Schneider, Tim Schröder, Anna Sefer, Piau Siong Tan, Johann Thurn, Philip Tinnefeld, John van Noort, Shimon Weiss, Nicolas Wendler, Niels Zijlstra, Anders Barth, Claus A. M. Seidel, Don C. Lamb, Thorben Cordes

**Affiliations:** 1 Department of Chemistry, Ludwig-Maximilians University München, München, Germany; 2 Physical and Synthetic Biology, Faculty of Biology, Ludwig-Maximilians University München, Planegg-Martinsried, Germany; 3 Molecular Physical Chemistry, Heinrich-Heine University Düsseldorf, Düsseldorf, Germany; 4 Department of Chemistry, University of Sheffield, Sheffield, UK; 5 B CUBE – Center for Molecular Bioengineering, Technische Universität Dresden, Dresden, Germany; 6 Department of Chemistry and Biochemistry, University of California, Los Angeles, CA USA; 7 Molecular Microscopy Research Group, Zernike Institute for Advanced Materials, University of Groningen, AG Groningen, the Netherlands; 8 Department of Biochemistry, Genetics and Microbiology, Institute of Microbiology, Single-Molecule Biochemistry Laboratory, University of Regensburg, Regensburg, Germany; 9 Department of Biophysics and Biophysical Chemistry, Johns Hopkins University School of Medicine and Howard Hughes Medical Institute, Baltimore, MD USA; 10 Dynamic Bioimaging Laboratory, Advanced Optical Microscopy Center and Biomedical Research Institute, Hasselt University, Agoralaan C (BIOMED), Hasselt, Belgium; 11 Department of Chemistry, KU Leuven, Leuven, Belgium; 12 Institute of Physics, University of Lübeck, Lübeck, Germany; 13 Institute of Physical Chemistry, University of Freiburg, Freiburg, Germany; 14 Signalling Research Centers BIOSS and CIBSS, University of Freiburg, Freiburg, Germany; 15 Department of Physics, Clarendon Laboratory, University of Oxford, Oxford, UK; 16 Kavli Institute of Nanoscience Discovery, University of Oxford, Oxford, UK; 17 Bayerisches NMR Zentrum, Department of Bioscience, School of Natural Sciences, Technical University of München, Garching, Germany; 18 Yusuf Hamied Department of Chemistry, University of Cambridge, Cambridge, UK; 19 Biocenter, Johannes Gutenberg University Mainz, Mainz, Germany; 20 Institute of Molecular Biology, Mainz, Germany; 21 Structural and Computational Biology Unit, European Molecular Biology Laboratory, Heidelberg, Germany; 22 Department of Biological Chemistry, The Alexander Silberman Institute of Life Sciences, and The Center for Nanoscience and Nanotechnology, Faculty of Mathematics and Science, The Edmond J. Safra Campus, The Hebrew University of Jerusalem, Jerusalem, Israel; 23 Centre de Biologie Structurale (CBS), University of Montpellier, CNRS, INSERM, Montpellier, France; 24 Biological and Soft Matter Physics, Huygens–Kamerlingh Onnes Laboratory, Leiden University, Leiden, the Netherlands; 25 Institute for Biophysics, Ulm University, Ulm, Germany; 26 Materials Science and Engineering, University of Illinois Urbana-Champaign, Urbana, IL USA; 27 Institute of Structural Biology, Molecular Targets and Therapeutics Center, Helmholtz Center Munich, Munich, Germany; 28 Cluster of Excellence Physics of Life, Technische Universität Dresden, Dresden, Germany; 29 California NanoSystems Institute, University of California, Los Angeles, CA USA; 30 Present Address: Warwick Medical School, The University of Warwick, Coventry, UK; 31 Present Address: Institute of Technical Physics, German Aerospace Center (DLR), Stuttgart, Germany; 32 Present Address: Department of Bionanoscience, Kavli Institute of Nanoscience, Delft University of Technology, Delft, the Netherlands

**Keywords:** Single-molecule biophysics, Molecular biophysics, Fluorescence resonance energy transfer, Proteins, Structure determination

## Abstract

Single-molecule Förster-resonance energy transfer (smFRET) experiments allow the study of biomolecular structure and dynamics in vitro and in vivo. We performed an international blind study involving 19 laboratories to assess the uncertainty of FRET experiments for proteins with respect to the measured FRET efficiency histograms, determination of distances, and the detection and quantification of structural dynamics. Using two protein systems with distinct conformational changes and dynamics, we obtained an uncertainty of the FRET efficiency ≤0.06, corresponding to an interdye distance precision of ≤2 Å and accuracy of ≤5 Å. We further discuss the limits for detecting fluctuations in this distance range and how to identify dye perturbations. Our work demonstrates the ability of smFRET experiments to simultaneously measure distances and avoid the averaging of conformational dynamics for realistic protein systems, highlighting its importance in the expanding toolbox of integrative structural biology.

## Abstract (web summary)

An international blind study confirms that smFRET measurements on dynamic proteins are highly reproducible across instruments, analysis procedures and timescales, further highlighting the promise of smFRET for dynamic structural biology.

## Main

Förster-resonance energy transfer (FRET) studies have become a widely used approach to complement classical structural biology techniques^1–4^. They provide information on the structure and conformational heterogeneity of biomolecules over a distance range of 30 to 120 Å and, when performed on single molecules, contribute additional information regarding conformational dynamics on the timescales of nanoseconds to seconds^1,2,5–10^. They also allow for quantitative assessment of structural dynamics and heterogeneity of conformational ensembles. This information is not easily accessible by X-ray crystallography, cryogenic-electron microscopy or cross-linking mass-spectrometry, which provide structural information of solution structures but lack temporal information. FRET can also be used to resolve (parts of) structures in an integrative manner (refs. ^11–17^) and has the unique ability to provide correlated information on structure and dynamics^1,2^.

Hellenkamp et al. presented a quantitative multilaboratory smFRET blind study assessing the validity of using smFRET for structural measurements. This study used static double-stranded DNA (dsDNA) that demonstrated a high reproducibility between the different laboratories with an uncertainty of ≤6 Å for the FRET-derived distances^18^. These results strongly supported the idea that standardized smFRET measurements in combination with standardized data analysis routines are a useful addition to the integrative modeling of static biomolecular structures^12,19,20^.

Here, we assessed whether the established procedures translate to more flexible biomacromolecules such as proteins that undergo conformational changes. Compared to dsDNA, proteins are more challenging systems, because the local environments and flexibility of the tethered dyes can vary considerably. Site-specific dye labeling of proteins usually requires the introduction of point mutations (for example, cysteines or nonnatural amino acids), which can affect its structure and function^1^. Moreover, proteins require careful handling and storage due to sample instability and aggregation, and are sensitive to experimental conditions, buffer composition, pH, temperature, surface interactions and so on. In a blind comparison study involving 19 laboratories using diffusion-based confocal smFRET, we investigated the maltose-binding protein (MalE) and the U2 Auxiliary Factor 2 (U2AF2), which display conformational dynamics on different time and length scales. We addressed two key questions: (1) how consistently can smFRET efficiency histograms (and the derived distances) be determined by different laboratories for protein samples prepared with stochastic fluorophore labeling? (2) How reliably can smFRET measurements detect structural dynamics in these proteins and what are the minimal structural fluctuations detectible?

Our study confirmed the reproducibility of accurate FRET efficiency histograms and the ability of smFRET to detect and quantify conformational dynamics on the submillisecond timescale. We demonstrate reproducible FRET efficiency values with uncertainties ≤0.06 corresponding to a distance precision of ≤2 Å and an accuracy ≤5 Å in MalE. Moreover, we compare the variability of setup-dependent parameters and identified the main sources of calibration uncertainty. To push the detection limits for structural dynamics, we refined established experimental and data analysis procedures and studied distinct dye pairs to identify and eliminate dye-specific effects. With this, we could detect distance fluctuations on the order of 5 Å in the FRET-sensitive range. Our work demonstrates that smFRET is able to characterize challenging and realistic protein systems with conformational dynamics on timescales from nanoseconds to seconds, highlighting its importance in the expanding toolbox of integrative structural biology^19–21^.

## Results

**Fig. 1:**
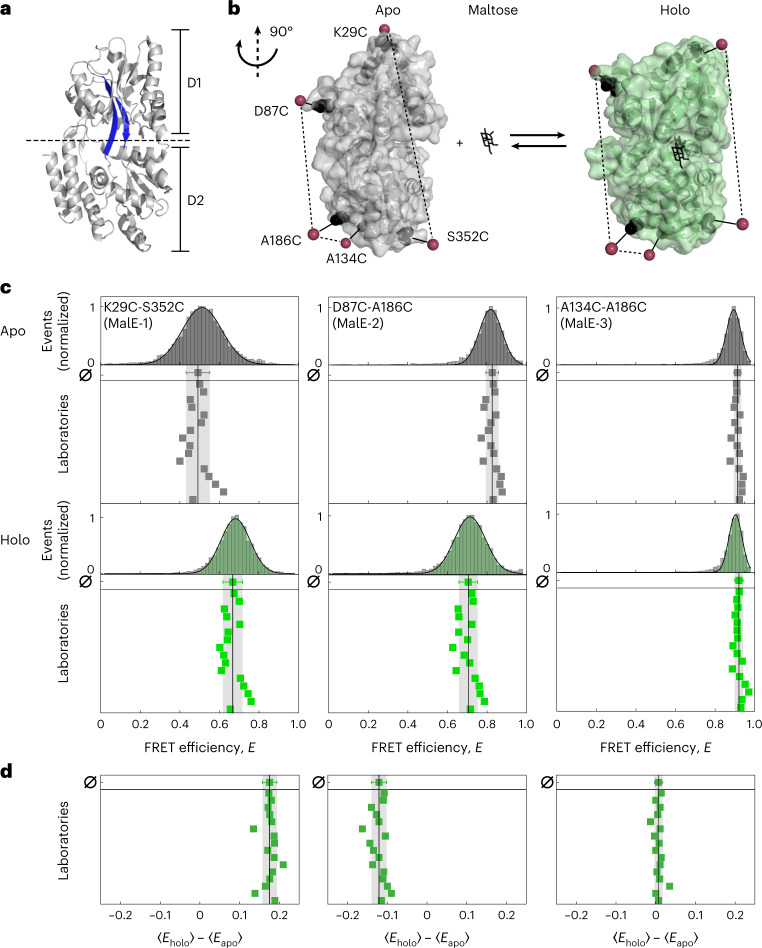
Experimental design of MalE as a protein model system for smFRET studies. **a**, Crystal structure of MalE in its ligand-free apo state (PDB ID 1OMP) with domains D1 and D2 linked by flexible beta sheets (highlighted in blue). **b**, The crystal structure of MalE (rotated by 90° as compared to **a** in the apo (gray, PDB ID 1OMP) and holo (green, PDB ID 1ANF) states with mutations at K29C-S352C (MalE-1), D87C-A186C (MalE-2) and A134C-A186C (MalE-3) indicated in black. Note, each mutant only contains one cysteine pair and was measured using the Alexa546–Alexa647 FRET pair. The estimated mean position of the fluorophores from AV calculations are shown as red spheres. **c**, FRET efficiency *E* histograms for three MalE mutants, MalE-1 (left), MalE-2 (middle) and MalE-3 (right), in the absence and presence of 1 mM maltose (bottom, green) for one exemplary dataset measured in laboratory 1. The distribution is fitted to a Gaussian distribution. The reported mean FRET efficiencies for 16 laboratories are shown below (due to experimental difficulties, the results of three laboratories were excluded; Supplementary Table 1). The mean FRET efficiency and the standard deviation of all 16 laboratories are given by the black line and gray area. **d**, Individual FRET efficiency differences for each laboratory, between the apo and holo states, $\left\langle {E_{{{{\mathrm{holo}}}}}}\right\rangle - \left\langle{E_{{{{\mathrm{apo}}}}}} \right\rangle$, for MalE-1 (left), MalE-2 (middle) and MalE-3 (right). The mean FRET efficiency difference and the standard deviation of all 16 laboratories are given by the black line and gray area. Source data

**Fig. 2:**
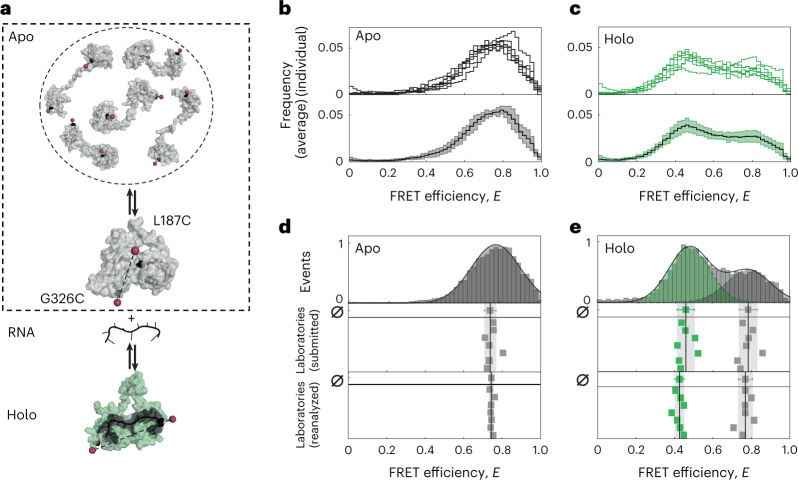
The experimental system of U2AF2 (RRM1, 2) and a comparison of FRET efficiency histograms from seven different laboratories. **a**, Schematic of the dynamics of U2AF2. The apo state (in gray, top) undergoes fast exchange between an ensemble of detached structures of which five representative structures are displayed. A slower exchange occurs between the dynamic detached ensemble and a compact conformation (PDB ID 2YHO) shown below. The holo state (in green, PDB ID 2YH1) bound to a U9 RNA ligand (in dark gray) assumes a well-defined, open conformation. Positions of cysteine mutations introduced for labeling (L187 in RRM1 and G326 in RRM2) are depicted as black spheres with the mean dye position determined by AV calculations indicated by red spheres. **b**,**c**, SmFRET efficiency histograms reported by the seven participating laboratories for apo (**b**) and holo (**c**) measurements of U2AF2. The top shows the individual FRET efficiency histograms and the bottom shows the average FRET efficiency histogram (solid line) with standard deviation (light area). **d**, SmFRET efficiency *E* histograms of U2AF2 in the apo state. The top shows a representative 1D FRET efficiency histogram with a Gaussian fit (laboratory 1). The middle shows the reported mean FRET efficiencies reported by seven laboratories. The mean value from all datasets is 0.739 ± 0.029, shown above with the corresponding standard deviation in gray. The bottom shows the extracted mean FRET values after reanalysis of the collected data. After reanalysis, the agreement improved to 0.742 ± 0.008. **e**, SmFRET efficiency histogram comparisons of U2AF2 in the holo state. 5 µM of U9 RNA was used to obtain the holo state. The top shows a representative 1D FRET efficiency histogram of laboratory 1 fitted to two Gaussian distributions to determine the FRET efficiencies of the different subpopulations, yielding mean FRET efficiencies of 0.44 for RNA-bound and 0.76 for the RNA-free conformation. The middle shows the mean FRET efficiencies reported by the seven laboratories. The mean values from all seven of the datasets were 0.45 ± 0.04 for the RNA-bound conformation (in green) and 0.78 ± 0.04 for the RNA-free conformation (in gray). The bottom shows the reanalysis of the holo measurements yielding values of 0.42 ± 0.02 and 0.77 ± 0.03 for RNA-bound and RNA-free fractions, respectively. Source data

We chose two protein systems with conformational dynamics on different timescales. Our first target was the MalE protein of *Escherichia coli*, the periplasmic component of the ATP binding cassette transporter MalFGK_2_-E (refs. ^22–24^). MalE exhibits a typical periplasmic-binding protein fold^25,26^ composed of two rigid domains connected by a flexible two-segment ß-stranded hinge (Fig. 1a). This structure enables an allosterically driven motion from an open to closed state upon maltose binding on the subsecond timescale (Supplementary Fig. 1). As a second system, we chose the large subunit of U2AF2 from the pre-messenger RNA (mRNA) splicing machinery^27^. Its two RNA recognition motif domains (RRM1,2) are connected by a long flexible linker and bind single-stranded Py-tract RNA^28^. For U2AF2, the two domains fluctuate between an ensemble of detached conformations and a compact conformation in the apo state^29^, whereas ligand binding stabilizes an open conformation (Fig. 2a)^30^.

SmFRET experiments were blindly performed by 19 laboratories for MalE and by seven laboratories for U2AF2 using different implementations of diffusion-based confocal spectroscopy with alternating excitation, that is, microsecond-ALEX (alternating laser excitation mode)^31^ for intensity-based analysis and nsALEX^32^ or pulsed-interleaved excitation (PIE)^33^ for intensity- and lifetime-based analyses (Supplementary Fig. 2). To avoid additional complexity and to restrict any preknowledge regarding the samples, the proteins were labeled and checked for functionality before being delivered to the participants. Information regarding the identity of the proteins and ligands, labeling positions, labeling efficiency, and expected FRET efficiencies and changes were not provided. The laboratories were informed about which fluorophores were coupled. We adapted a data analysis routine similar to ref. ^18^ to determine setup-independent accurate FRET efficiency *E* values from the photon counts detected in the donor (D) and acceptor (A) detection channels during a single-molecule event. The procedure is described in the Methods and includes subtraction of background signals from all channels and the determination of four correction factors: (*α*) for spectral crosstalk of D fluorescence into the A channel, (*β*) for normalization of direct D and A excitation fluxes, (*γ*) for differences in D and A quantum yields and detection efficiencies and (*δ*) for the ratio of indirect and direct A excitation (Supplementary Fig. 3 and Supplementary Tables 1 and 2)^34^. The use of ALEX or PIE (Supplementary Note 1) was crucial for corrections of the photon counts to reflect the actual D and A signal and exclusion of single-molecule events from incompletely labeled molecules or ones showing photo- blinking and bleaching^8,9,18,34,35^.

### MalE

We prepared three double-cysteine variants of MalE with interresidue distances that cover a large part of the dynamic range of FRET (Fig. 1b, Methods and Supplementary Fig. 4). The variants were designed to show a decrease (MalE-1, K29C-S352C), an increase (MalE-2, D87C-A186C) or an unaltered interdye distance (MalE-3, A134C-A186C) upon maltose binding. All variants of MalE were stochastically labeled in one of the laboratories at the given positions with the donor Alexa Fluor 546 (Alexa546) and acceptor Alexa Fluor 647 (Alexa647). Before shipment, we confirmed the functionality of the labeled protein by ligand titrations using smFRET and microscale thermophoresis, and verified that maltose did not affect the dye properties (Supplementary Figs. 5 and 6). To allow a comparison, participants were asked to provide mean FRET efficiencies using Gaussian fits for apo and holo FRET efficiency histograms (Fig. 1c) and to determine a global *γ* value for all measurement conditions (Supplementary Note 2 and Supplementary Fig. 3). For this workflow, participants used custom or publicly available software packages.

FRET efficiency histograms for representative experiments on MalE in the apo (no ligand) and the holo state (1 mM maltose) are shown in Fig. 1c with mean values reported by 16 laboratories. They show very good agreement and reproducibility. It was not possible to extract accurate FRET efficiency values from three laboratories due to, for example, missing or suboptimal laser lines (Supplementary Table 1 and Supplementary Note 2). All laboratories observed the expected changes for MalE-1, MalE-2 and no shift for MalE-3. This indicates that the samples did not degrade during shipment on dry ice and storage in the laboratories at 4 °C. MalE-1 showed an average FRET efficiency of 0.49 ± 0.06 in the apo state that increased to 0.67 ± 0.05 in the holo state. MalE-2 showed the expected decrease in FRET efficiency from 0.83 ± 0.03 to 0.71 ± 0.05 in the apo and holo states, respectively (Fig. 1c). MalE-3, with both labels on one lobe, showed no significant change in FRET efficiency (*E*_apo_ = 0.91 ± 0.02, *E*_holo_ = 0.92 ± 0.02).

The standard deviation of the determined mean FRET efficiency over all laboratories was less than ±0.06, similar to the precision found for dsDNA previously^18^ (Extended Data Table 1 and Supplementary Table 3). We observe the highest standard deviation for MalE-1 and the lowest values of ±0.02 for MalE-3, which also has the highest FRET efficiency. We observed systematic deviations of the reported FRET efficiency values for the apo and holo states from the mean value. Hence, we analyze the individual FRET efficiency differences, $\left\langle {E_{{{{\mathrm{holo}}}}}}\right\rangle - \left\langle{E_{{{{\mathrm{apo}}}}}} \right\rangle$, between the apo and holo states for the different laboratories (Fig. 1d). The distributions indeed narrow for all samples by approximately twofold because systematic deviations cancel out (${\sigma}_{\left\langle {E_{{{{\mathrm{holo}}}}}}\right\rangle - \left\langle{E_{{{{\mathrm{apo}}}}}} \right\rangle }$ for MalE-1 ±0.02, MalE-2 ±0.02 and MalE-3 ±0.01: Fig. 1d, Extended Data Table 1 and Supplementary Table 3).

### U2AF2

For the second protein, U2AF2, we chose the published double-cysteine variant L187C-G326C of the minimal RRM1,2 construct, where we previously verified that protein function is not affected by labeling (Fig. 2a)^36,37^. The construct was labeled stochastically on the two RRM domains with the dye pair Atto532*–*Atto643. A subset of seven groups measured the sample. To investigate the consistency of the obtained FRET efficiency histograms, we plotted the smFRET histograms from individual laboratories (Fig. 2b,c, row 1) as well as the average distribution illustrated by the mean and standard deviation (row 2). All groups found a single broad distribution (Fig. 2b, row 1, apo) with an average *E* = 0.74 ± 0.03 (row 2). In the presence of 5 µM ligand (U9 RNA, *K*_d_ of roughly 1.3 µM), a second narrower peak at lower *E* appears (Fig. 2c, row 1) with an average *E* = 0.46 ± 0.04 (row 2) as expected for the open conformation of the holo state^30,36^. Notably, a fraction of around 15% of ligand-free protein remains in the sample at the RNA concentration used (Supplementary Fig. 7).

For the apo state, we obtained a similar standard deviation of ±0.03 as found for MalE, however, a clear outlier was apparent (Supplementary Table 4). To test whether user bias affected the reported results, a single person reanalyzed the datasets. This person developed an optimal procedure for determining the correction factors for this challenging sample (Supplementary Note 3) and improved the agreement to a standard deviation of ±0.008 with no change in mean *E* (Fig. 2d,e and Supplementary Table 4). The reanalysis revealed the detection correction factor *γ* to be the main cause of the deviations between the measurements. As a single population of the apo state did not allow for a robust determination of the *γ* factor^34,35^, it was best to estimate the *γ* factor from a global analysis of the apo and holo measurements. This was possible since the quantum yield of the fluorophores remained unchanged upon RNA binding (Supplementary Table 5). We also reanalyzed data from the same seven laboratories for MalE-1 apo and obtained nearly identical mean FRET efficiencies and standard deviations (0.49 ± 0.05 versus 0.47 ± 0.06, Supplementary Fig. 8). This indicates that user bias was less pronounced when a global, well-defined analysis procedure for determining *γ* was provided over several samples covering a substantial fraction of the FRET range (Supplementary Note 2).

For the holo state of U2AF2, good agreement between laboratories was obtained for the peak positions with a standard deviation of ±0.03 and ±0.02 for the high- and low-FRET peaks, respectively. A minimal improvement resulted from the reanalysis (Supplementary Table 4). In contrast to the agreement in FRET efficiency, we observed variations in the relative amplitudes of the two populations: 0.58 ± 0.08 for the holo state and 0.42 ± 0.08 for the apo population (Fig. 2c and Supplementary Table 4). We attribute this to potentially reduced protein activity, degradation of the RNA ligand and sensitivity of conformational dynamics to the experimental conditions, for example, temperature, ligand concentration, buffer composition, salt concentration or the presence of stabilizers such as bovine serum albumin (BSA) (Supplementary Fig. 7).

### Setup-dependent parameters and correction factors

**Fig. 3:**
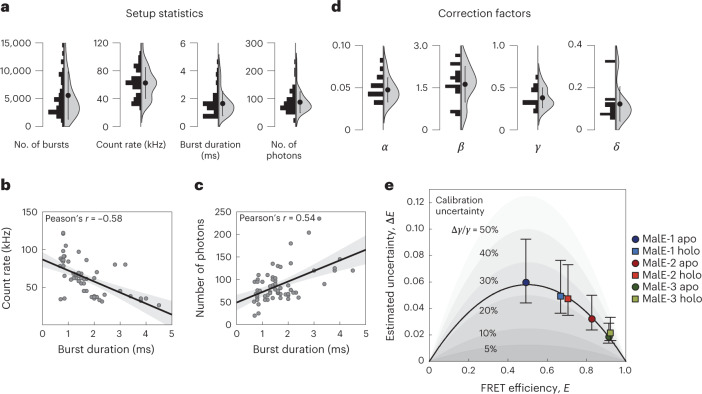
Setup-dependent parameters and calibration uncertainty. **a**, The distribution of the parameters quantifying the statistics of the measurements and the performance of the setups used for both MalE and U2AF2 measurements are shown as histograms and violin plots for the measurements from eight laboratories. The circle and whiskers in the violin plot indicate the mean and standard deviation (*n* = 64, averaged over eight samples measured in the eight different laboratories). Sample-dependent distributions of the shown parameters are given in Supplementary Fig. 9. **b**,**c**, Pairwise plots of the average count rate (**b**) and the number of photons (**c**) against the burst duration. The same datasets are plotted as used for **a**. While the count rate decreases slightly for longer burst durations, a positive correlation is observed for the acquired number of photons per burst and the burst duration, indicating that larger observation volumes result in a higher accumulated signal per molecule. Correlations between all parameters are shown in Supplementary Fig. 10. Error bands indicate the 95% confidence intervals of the regression. **d**, The distributions of the four correction factors for the calculation of accurate FRET efficiencies for all the MalE measurements are shown as histograms and violin plots for the measurements from all laboratories. The circle and whiskers in the violin plot indicate the mean and standard deviation (*n* = 64, averaged over eight samples measured in the eight different laboratories). **e**, A plot of the standard deviation of the reported FRET efficiencies from 16 laboratories (as a measure of the experimental uncertainty) against the average FRET efficiency for the MalE mutants 1–3 reveals that lower uncertainties are observed for higher FRET efficiencies. The black line represents a fit of the estimated uncertainties under the assumption that the variations arise solely due to uncertainty in the *γ* factor (equation (1)). The inferred relative uncertainty of the *γ* factor is around 23%. Shaded areas indicate relative uncertainties of 5–50%. Error bars indicate 95% confidence intervals around the average value. Source data

The quality of smFRET experiments is determined by the statistics of the measurement and the performance of the setup to maximize photon collection and thereby minimize shot noise. To this end, we quantified the number of bursts, average photon count rate, burst duration and the number of photons in the D and A channels for the MalE measurements from eight laboratories (Fig. 3a and Supplementary Fig. 9). On average, participants collected 6,000 bursts (minimum 500, maximum 21,000) of molecules carrying both fluorophores. The required number of bursts for a smFRET analysis depends on the goal of the experiment. To determine the average FRET efficiency from a single population, as performed for MalE, roughly 1,000 bursts of double-labeled molecules may be sufficient. For advanced analysis methods such as time-correlated single photon counting (TCSPC) for lifetime analysis, burst-wise fluorescence correlation spectroscopy (FCS) or a photon distribution analysis (PDA) that are applied to subensembles, higher burst numbers of >5,000 are desired. Typical count rates per single-molecule event were found to be 60 ± 20 kHz, with an average burst of 90 ± 40 photons and 1.7 ± 0.9 ms duration (Fig. 3a and Supplementary Fig. 9). The average count rate and burst duration depend on the size of the confocal volume, where smaller sizes result in higher count rates but shorter burst durations. We observe a negative correlation between burst duration and average count rate (Fig. 3b, Pearson’s *r* = −0.58 and Supplementary Fig. 10). The large spread of the burst duration arises from the fact that some participants applied a diffraction-limited observation volume, while others underfilled the objective lens to create a larger confocal volume with a diameter of roughly 1 µm (assuming that the detection pinhole corresponds to the excitation volume). We also observed a small positive correlation between detected photon numbers and burst duration (Fig. 3c, Pearson’s *r* = 0.54 and Supplementary Fig. 10). This suggests that larger volumes, in combination with high irradiances, yield the highest number of photons per burst^38^. Smaller volumes generally allow for higher burst collection rates with higher count rates and thus shorter interphoton times, enabling fast transitions on the sub-µs timescale to be resolved^39,40^. Longer burst durations offer the benefit that slower dynamics can be studied.

For an accurate analysis, the correction factors for donor spectral crosstalk (*α*), excitation flux (*β*), detection efficiency and quantum yields (*γ*) and direct acceptor excitation (*δ*) must be determined (see ref. ^18^, Supplementary Table 5). We plot the distribution of the correction factors used to determine accurate FRET efficiencies for MalE in Fig. 3d from 16 laboratories (Supplementary Table 1). Besides fluorophore properties, these also depend on setup-specific parameters including dichroic mirrors, emission filters, detectors, excitation wavelengths and laser power. Nonetheless, we observed a well-defined distribution for *α* of 0.05 ± 0.01, which is determined by the emission filters and detectors in both detection channels. A larger spread was observed for *β* values of 1.6 ± 0.6 and *δ* of 0.12 ± 0.08. These depend on the ratio of the excitation powers, where most participants used about half the laser power for direct acceptor excitation (45 ± 27 µW) in comparison to the donor excitation (78 ± 58 µW), resulting in similar count rates after donor and acceptor excitation. The agreement between the reported FRET efficiency values clearly shows that the diverse experimental settings are compensated by the correction procedure applied here.

For *γ*, which is the most difficult factor to determine, we observed an average of 0.4 ± 0.1 (Supplementary Fig. 3b). It depends on the acceptor-to-donor ratio of the detection efficiencies, *g*, and the effective fluorescence quantum yields, *ϕ*_F_, as $\gamma = g_{\mathrm{A}}\phi _{{\mathrm{F,A}}}/g_{\mathrm{D}}\phi _{{\mathrm{F,D}}}$ (ref. ^18^). Similar to crosstalk, *γ* strongly depends on the emission filters and the type of detectors used. Due to *ϕ*_F,A_ of roughly 0.32 (acceptor) and *ϕ*_F,D_ of roughly 0.72 (donor), all laboratories reported *γ* factors below 1. Despite the large spread in the reported values, we observed very good agreement for the reported FRET efficiencies in our blind study. Our analysis identified *γ* as the key factor limiting the consistency, which is supported by the following arguments: (1) in Fig. 1d, the spread of $\left\langle {E_{{{{\mathrm{holo}}}}}}\right\rangle - \left\langle{E_{{{{\mathrm{apo}}}}}} \right\rangle$ is smaller (for example, 0.06 to 0.02 for MalE-1) than for absolute *E* values in Fig. 1c, suggesting that errors in *E* are systematic rather than random. (2) The observed spread in reported FRET efficiencies depends on the absolute FRET efficiency measured for MalE (Fig. 1c,d). (3) We also calculated the uncertainty in the FRET efficiency calculation using error propagation for crosstalk, direct excitation and background correction in the donor and acceptor channels. The reported uncertainty can be attributed mainly to the *γ* factor (Fig. 3e and Supplementary Note 4) with the error of the *γ* factor, Δ*γ*, that propagates into an uncertainty in the reported FRET efficiencies, Δ*E*:

1 $$\begin{array}{*{20}{c}} {\Delta E = E\left( {1 - E} \,\right)\frac{{{{\Delta }}\gamma }}{\gamma }} \end{array}$$

Notably, the observed experimental Δ*E* is well described by equation (1) (black line in Fig. 3e), yielding a relative uncertainty of Δ*γ*/*γ* = 23% corresponding to Δ*γ* ≅ 0.07. The improved agreement between measurements on reanalysis for U2AF2 (Fig. 2d) suggests that the accuracy of the analysis could be improved by standardized procedures for the determination of all correction factors, which differ depending on the number of populations in the measurement and whether the FRET efficiency peak is dynamically averaged (Supplementary Note 2).

### Detection and quantification of conformational dynamics

Fluorescence trajectories of immobilized molecules provide access to kinetics on the millisecond to second timescales via a dwell-time analysis (Supplementary Fig. 1)^41–43^. For freely diffusing molecules, millisecond dynamics can be studied in the same fashion when molecules diffuse slowly^44,45^. The detection and quantification of submillisecond conformational dynamics in quickly diffusing molecules (with the maximum timescale limited by the burst duration) is possible via FRET–FCS^44,46,47^, filtered-FCS^48,49^, burst-variance analysis (BVA)^50^, FRET–two-channel kernel-based density distribution estimator^51^, dynamic PDA^52^, FRET efficiency *E* versus fluorescence-weighted average donor lifetime $\left\langle {\tau _{{\mathrm{D(A)}}}} \right\rangle _{\mathrm{F}}$ analysis (*E*–τ plots)^52,53^, nanosecond-FCS^54^, recurrence analysis of single particles^55^, photon-by-photon maximum likelihood approaches^40,56–59^ and Monte Carlo diffusion-enhanced photon inference (MC-DEPI)^60^. To assess how consistently dynamics can be detected in smFRET measurements, we asked the participants to evaluate whether the proteins were static or dynamic on the (sub-)millisecond timescale and which method they used to come to this conclusion (Supplementary Table 6).

**Fig. 4:**
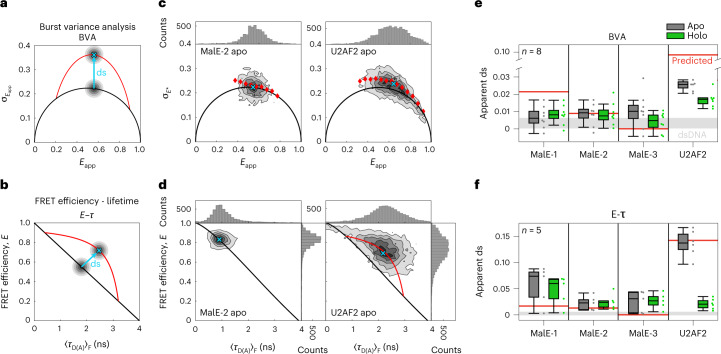
Detection and characterization of conformational dynamics on the submillisecond timescale in MalE and U2AF2. **a**,**b**, Schematic representations of BVA (**a**) and *E*–τ (**b**) plots. The ds is defined as the excess standard deviation compared to the static line (shown in black). Dynamic FRET lines are indicated in red. **c**, BVA of MalE-2 labeled with Alexa546–Alexa647 without maltose (apo, left) and U2AF2 labeled with Atto532–Atto643 without RNA (apo, right). Here, the BVA is based on a photon binning of five photons. Red diamonds indicate the average standard deviation of all bursts within a FRET efficiency range of 0.05. The mean positions of the populations (cyan crosses) were determined by fitting a two-dimensional Gaussian distribution to the data (Supplementary Note 5). **d**, The plots of the FRET efficiency *E* versus intensity-weighted average donor lifetime $\left\langle {\tau _{{\mathrm{D(A)}}}} \right\rangle _{\mathrm{F}}$ of the same measurement as in **c**. The donor-only population was excluded from the plot. For MalE-2, the population falls on the static FRET line, while a clear ds is observed for U2AF2. The endpoints of the dynamic FRET line for U2AF2 were determined from a subensemble analysis of the fluorescence decay. **e**,**f**, The apparent ds of the peak of the population was determined graphically from BVA (eight laboratories for MalE and seven laboratories for U2AF2, respectively) (**e**) and *E*–τ (five laboratories) (**f**) plots (Methods). For U2AF2 in the holo state, the ds was assessed only for the low-FRET RNA-bound population. Boxes indicate the median and 25/75% quartiles of the data. Whiskers extend to the lowest or highest data point within 1.5 times the interquartile range. The gray area indicates the ds obtained for the dsDNA used in a previous study^18^ based on measurements performed in laboratory 1 for BVA (ds_DNA_ = 0.0033 ± 0.0033) and laboratory 2 for the *E*–τ plot (ds_DNA_ = 0.0026 ± 0.0044). The horizontal red lines indicate the expected ds for a potential conformational exchange between the apo and holo states. We computed the expected change in FRET efficiency using their structural models in the PDB (Supplementary Note 6 and Supplementary Table 9). Source data

BVA and *E*–τ plots are frequently used techniques to visualize FRET dynamics by comparing the measured data to theoretical expectations. BVA detects dynamics by estimating the standard deviation of the FRET efficiency over individual bursts, using a predefined photon window (typically ≳100 µs depending on the molecular brightness). Due to FRET dynamics, the standard deviation of the FRET signal within a burst (red line in Fig. 4a) can be higher than expected from shot noise (black semicircle in Fig. 4a), which becomes visible as a deviation or apparent dynamic shift, ds^50^. In the *E*–τ plots, the observed FRET efficiency determined via intensity (Fig. 4b) is a species-weighted average and, in the presence of dynamics, the position along the *y* axis depends on the fraction of time spent in the respective states. The fluorescence lifetime of the donor (Fig. 4b, $\left\langle {\tau _{{\mathrm{D(A)}}}} \right\rangle _{\mathrm{F}}$, *x* axis) is a photon-weighted average, because only a single lifetime is determined. Hence, it is weighted toward the lifetime of low-FRET states as they emit more donor photons^52,53^, shifting the data to the right of the ‘static’ FRET line. *E*–τ plots can detect dynamics on the nanosecond to millisecond timescale. Here, we have included an additional correction that considers distance fluctuations of the flexible dye linkers (6 Å) resulting in a slightly curved ‘static’ FRET line^52,61^. To quantify dynamics between two distinct states, a theoretical ‘dynamic’ FRET line (red, Fig. 4b) is overlaid. Again, ds is defined as the deviation of the observed data from the theoretical static line (Fig. 4b and Supplementary Note 6). It is important to mention that FRET dynamics, and the related ds, are not always of conformational origin.

MalE exhibits slow ligand-driven dynamics on the subsecond timescale between high- and low-FRET states (Supplementary Fig. 1)^62^. Here, we investigated whether the apo and/or holo states undergo dynamics faster than the timescale of diffusion. Both techniques reveal that MalE exhibits no large FRET-fluctuations on the ms timescale (Fig. 4c,d and Supplementary Fig. 11). Almost all groups confirmed this assessment and only three groups concluded that MalE is dynamic without further justification (Supplementary Table 6). To investigate the presence of potential dynamics in more detail, we determined the ds for a subset of the data (eight laboratories for BVA, Fig. 4e and five for *E*–τ, Fig. 4f, Supplementary Note 5, and Supplementary Table 7). As a static control, we determined the ds of the dsDNA rulers used in ref. ^18^ (mean ± 1 s.d. as determined from laboratories 1 and 2) shown in gray in Fig. 4e,f (Supplementary Table 8). The ds did not exceed that of the static reference when using BVA for all MalE mutants. From the *E*–τ plots, however, ds was higher than for dsDNA, especially for MalE-1. This sample clearly exceeds what is predicted for a static system or even what is predicted for dynamics between the apo and holo states (Fig. 4f, red lines and Supplementary Note 6). Hence, some laboratories categorized MalE as dynamic. The cause of this ds, which must originate from FRET dynamics that are faster than around 100 µs, will be discussed in detail below.

In contrast to MalE, all groups found U2AF2 to be dynamic as was expected for two domains connected by a flexible linker (Fig. 4c–f and Supplementary Table 6). The ligand-free apo state shows pronounced deviations from the behavior for static molecules both in the BVA and *E*–τ plots, while the RNA-bound holo state shows a notable ds for BVA but not for the *E*–τ analysis (Fig. 4c–f). It was challenging to assess whether the holo state is truly static or dynamic since it contained a measurable fraction of apo protein, which overlaps with the holo population. Hence, U2AF2 is a challenging test case, yet, dynamics were unambiguously detected in all laboratories demonstrating the reliability of smFRET for investigating dynamic systems.

### Accuracy of FRET-derived distances and structural modeling

**Fig. 5:**
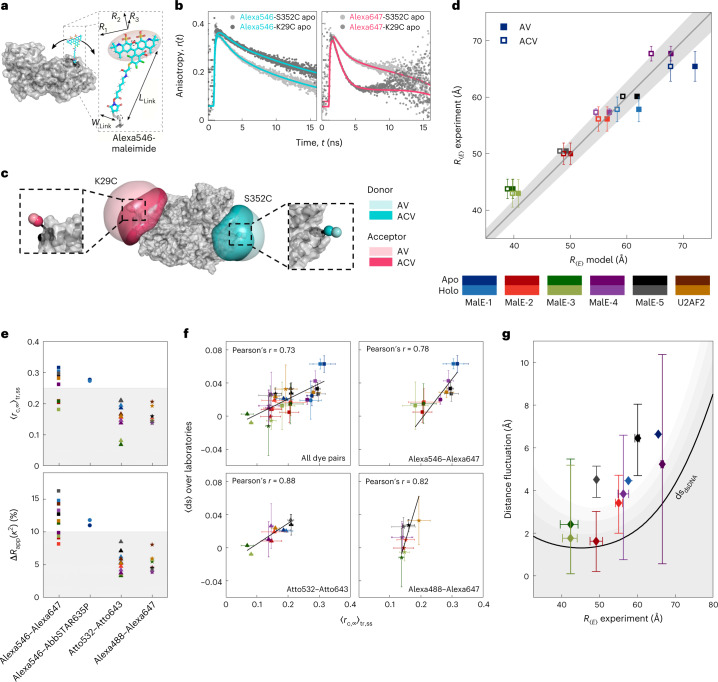
Assessing the accuracy of smFRET-derived distances in MalE. **a**–**d**, AV calculations and model-based interdye distances. **a**, Schematic of Alexa546 attached to MalE (PDB 1OMP) showing the parameters needed for the AV calculations using the AV3 model^6^ (Supplementary Table 10). **b**, Fluorescence anisotropy decays of single-cysteine mutants for the donor (Alexa546, left) and acceptor (Alexa647, right) at the labeling positions K29C and S352C. Solid lines represent fits to a model with two or three rotational components (Supplementary Tables 11 and 12 and Supplementary Note 8). **c**, AV (light color) and ACV (dark color) calculations for Alexa546 (cyan) and Alexa647 (pink) at labeling positions 352 and 29. The zoom-ins show the mean positions of the dyes based on the AV (light shade) and ACV (darker shade) models. **d**, Comparison of the experimentally obtained FRET-averaged distance $R_{\left\langle E \right\rangle }$ with the theoretical model distances using the AV (filled squares) and ACV (empty squares) calculations. Errors represent the standard deviation in experimental distances (*n* = 16 laboratories for MalE mutants 1–3, *n* = 2 laboratories for MalE mutants 4–5, *n* = 7 laboratories for U2AF). The solid line represents a 1:1 relation and the gray area indicates an uncertainty of ±3 Å for a Förster radius of *R*_0_ = 65 Å. MalE-4 and -5 were measured by two laboratories. **e**, Detection of dye-specific protein interactions. Top shows the five MalE mutants and U2AF2 labeled with different dye combinations to determine the donor–acceptor-combined residual anisotropy, 〈*r*_c,∞_〉_tr,ss_ (*n* = 3 laboratories). Bottom shows the distance uncertainty relating to *κ*^2^, ${{\Delta }}R_{{{{\mathrm{app}}}}}\left( {\kappa ^2} \right)$, estimated (Supplementary Note 8). A maximum allowed distance uncertainty of ≤10% (shaded gray region) in ${{\Delta }}R_{{{{\mathrm{app}}}}}\left( {\kappa ^2} \right)$ leads to a dye-independent threshold of 0.25 for 〈*r*_c,∞_〉. **f**, The apparent dynamic shift 〈ds〉 versus the combined residual anisotropy 〈*r*_c,∞_〉 is shown for all measured dye pairs (top left) and individually. Error bars of the apparent ds represent the standard deviation over *n* = 3 laboratories. For the combined residual anisotropy, the propagated 1*σ* uncertainty (Supplementary Note 8). **g**, The structural flexibility of MalE estimated after filtering using the distance uncertainty threshold shown in **e** (Supplementary Note 12). Error bars represent the 1*σ* percentiles averaged over all dye pairs (*n* = 1, MalE-1; *n* = 7, MalE-2 and MalE-3; *n* = 4, MalE-4 and *n* = 5, MalE-5). The residual distance fluctuations obtained from control measurements on dsDNA in one laboratory (ds_dsDNA_ = 0.0026 ± 0.0044) are shown as a black line (gray areas represent confidence intervals of 1*σ*, 2*σ* and 3*σ*). Source data

Accurate FRET efficiencies need to be converted into distances for comparison with structures or to use them as distance constraints in integrative FRET-assisted structural modeling^1,5,7,15,63,64^. SmFRET experiments yield FRET efficiencies as a result of dynamically, nonlinearly averaged distances due to the flexible fluorophore linkers. To assess the accuracy of our measurements, we applied the accessible volumes (AV) approach^5,6,64,65^, which uses a coarse-grained dye model to estimate the FRET efficiency averaged model distance $R_{\left\langle E \right\rangle }^{{{{\mathrm{model}}}}}$ between the two dyes. For this, all possible positions of the fluorophores are averaged, taking into account linker conformations and steric hindrances (Fig. 5a–c, Methods and ref. ^6^). For AV calculations, we assume fast rotational and slow positional averaging with respect to the fluorescence lifetime. Prediction of measured distances via FRET values based on the flexibility and attachment points of a fluorophore is an area of active research and alternative methods are being developed, for example, rotamer libraries^66^ or molecular dynamics simulations^67,68^.

The average experimental FRET efficiencies from the individual smFRET histograms $\left\langle E \right\rangle$ for MalE (Fig. 2) were used to determine $R_{\left\langle E \right\rangle }$ for each laboratory (Extended Data Table 1 and Supplementary Table 3) using the Förster equation (equation (2)):

2 $$\begin{array}{*{20}{c}} {R_{\left\langle E \right\rangle} = R_0\left( {\frac{1}{{\left\langle E \right\rangle }} - 1} \right)^{\frac{1}{6}}} \end{array}$$

The Förster radius of Alexa546–Alexa647 on MalE was determined to be *R*_0_ = 65 ± 3 Å (Supplementary Note 7). Figure 5d displays the correlation between the experimental observable $R_{\left\langle E \right\rangle }$ and predicted $R_{\left\langle E \right\rangle }^{{{{\mathrm{model}}}}}$ using apo and holo structures exhibiting an uncertainty of 3–5 Å over all variants. In agreement with the predictions by Peulen et al.^69^, this accuracy is achieved despite stochastic protein labeling, which could result in different charge environments and AVs of the fluorophores depending on the labeling positions. This is evident by the varying dye behavior at different locations (Fig. 5b). During the study, three laboratories studied additional MalE variants (MalE-4, K34C-N205C and MalE-5, T36C-N205C) with a larger FRET efficiency contrast between the apo and holo states, complementing the results of the other variants (Extended Data Table 1).

Figure 5d reveals the largest deviation between experimental and predicted distances for MalE-1, which also had the highest ds values (Fig. 4f and Supplementary Fig. 11). Therefore, we investigated the role of dye*–*protein interactions using single-cysteine variants of MalE by measuring the fluorescence lifetimes, and time-resolved and steady-state anisotropies (Supplementary Note 8, Supplementary Tables 5, 11 and 12 and Fig. 5b). Labeling at residue 352 promotes dye sticking to the protein surface indicated by multiexponential fluorescence lifetimes and a high residual anisotropy, *r*_*∞*_, for both fluorophores (*r*_*∞*_ > 0.25). Labeling at residue 29 only shows sticking for the donor (*r*_*∞*,D_ > 0.30, *r*_*∞*,A_ roughly 0.12). At other positions (for example, residue 186), free rotation is possible for both dyes (Supplementary Tables 5 and 11). These position-specific interactions can cause the observed deviations between the experiment and structural model (Fig. 5d and Extended Data Table 1) and high ds values for MalE-1 (Fig. 4f). By using the accessible contact volume (ACV) approach^63^, which accounts for dye*–*protein interactions, the root-mean-average deviation between the structural model and experimental values decreased from 3 Å for AV to 2 Å (Fig. 5c). For protein labeling on opposite sides, dye*–*protein interactions in the ACV model result in reduced model distances and improved accuracy for all outliers (Fig. 5d and Extended Data Table 1).

It was suggested to use the combined residual anisotropy of D and A ($r_{{\mathrm{c}},\infty } = \sqrt {r_{\infty ,{\mathrm{D}}} \; r_{\infty ,{\mathrm{A}}}}$) for filtering out dye-related artifacts in FRET-assisted structural modeling with an empirical threshold of *r*_c,*∞*_ < 0.2 (refs. ^13,70^). To further investigate dye-specific sticking, three laboratories studied MalE mutants with the additional dye pairs Alexa546–AbbSTAR635P, Atto532–Atto643 and Alexa Fluor 488 (Alexa488)–Alexa647 and determined the residual anisotropies and distance uncertainties based on the orientation factor *κ*^2^ (Fig. 5e, top, Supplementary Tables 13 and 14 and Supplementary Notes 8 and 9). The dye pair Alexa546–Alexa647 showed the highest combined anisotropies (Supplementary Fig. 12a and Supplementary Table 13), which is attributed to the donor Alexa546 as the combined anisotropy also remains high for Alexa546–AbbSTAR635P but is reduced for Alexa488*–*Alexa647. To derive a robust and well-defined threshold for recognizing measurements with dye artifacts, we determined the uncertainty in the FRET-derived distances, Δ*R*_app_(*κ*^2^), that originates from the uncertainty of the orientation factor *κ*^2^. Previous approaches estimated the uncertainty in *κ*^2^ from the residual anisotropy in terms of rotational restrictions (wobbling-in-a-cone model)^70–73^. Here, we used a ‘diffusion with traps’ model, which assumes two dye populations (free and trapped) and relates the residual anisotropies to the fraction of dyes interacting with the surface of the biomolecule (Supplementary Note 9). Based on the estimated distance uncertainty, we propose a threshold of Δ*R*_app_(*κ*^2^) < 10% to identify measurements with dye-related artifacts (Fig. 5e, bottom). This threshold corresponds to a combined residual anisotropy of 0.25, similar to the previously suggested empirical threshold value of around 0.2 (refs. ^13,70^).

Next, we investigated whether dye sticking could cause the ds in the *E*–τ plot for MalE-1 with Alexa546–Alexa647 (Fig. 4f). To be observable in the *E*–τ plot, the exchange between the free and trapped dye species must occur faster than the diffusion time of roughly 1 ms, otherwise the two species would be observable as individual peaks. We observed a correlation between the laboratory-averaged 〈ds〉 and 〈*r*_c,*∞*_〉 over all dye pairs (Pearson’s *r* = 0.73), with a stronger correlation when each dye pair is investigated individually (Fig. 5f). As conformational dynamics should be label independent, dye sticking is likely responsible for the observed ds values. The *x* intercept of the linear fit is between 0.1 and 0.2, suggesting a dye-dependent anisotropy threshold needs to be considered. When applying the criteria 〈*r*_c,*∞*_〉 < 0.25 to MalE-1 (Supplementary Fig. 12b), only the dye pair Atto532–Atto643 should be used for distance determination, which also showed a markedly reduced ds (Supplementary Fig. 12c). Lifetime analysis of MalE-1 donor-only molecules showed donor quenching only at position 352, which confirms that labeling at this position is problematic (Supplementary Fig. 12c, Supplementary Note 10 and Supplementary Table 5).

Using the above criteria of 〈*r*_c,*∞*_〉 < 0.25 to minimize the influence of dye artifacts on the ds, we hypothesized that the remaining ds could be indicative of low-amplitude, fast conformational fluctuations. A *P* test analysis between the ds for dsDNA and protein samples (*P* < 0.05) indicated that the ds is still significant for various protein variants after filtering out dye artifacts (Supplementary Note 11, Supplementary Table 8). To estimate the conformational fluctuations necessary to generate the observed ds (Fig. 4f and Supplementary Table 8), we assume that dynamics occur between two nearby states with interdye distances of $R_{\left\langle E \right\rangle } \pm \delta R$ where δ*R* is the amplitude of the fluctuation^61^ (Fig. 5g, Supplementary Note 12 and Supplementary Table 8). This inferred fluctuation provides an upper bound for the conformational flexibility because factors such as calibration errors, dye blinking or photoisomerization could contribute to the observed ds. We consider the ds obtained from dsDNA as the lower limit (black line in Fig. 5g, ds_DNA_ = 0.0026 ± 0.0044: Supplementary Note 12), which defines the current detection limit for dynamics in smFRET experiments. The MalE variants 1, 4 and 5 exceed the ds for dsDNA by 2–3 Å (Fig. 5g, Supplementary Fig. 13 and Supplementary Table 8). Consistent with the smFRET results, all-atom molecular dynamics simulations of MalE using the ff14SB force field^74^ (Supplementary Note 13) suggest thermally induced conformational fluctuations with a standard deviation up to roughly 3 Å at the labeled residues in MalE-1, MalE-4 and MalE-5. This is larger than the typical fluctuations of about 1 Å (ref. ^75^) and leads to a broadening of the interresidue distance distributions for these FRET pairs. We conclude that the observed ds in the experiments can be explained by a combination of measurement uncertainty and small-scale structural fluctuations. Note that such small-scale fluctuations can be amplified in FRET experiments when the dye linker acts as a lever arm for appropriate labeling positions. A detailed discussion of the theoretical limits for detecting dynamics in smFRET experiments using BVA or the *E–*τ is given in Supplementary Note 14.

### Quantitative analysis of U2AF2

**Fig. 6:**
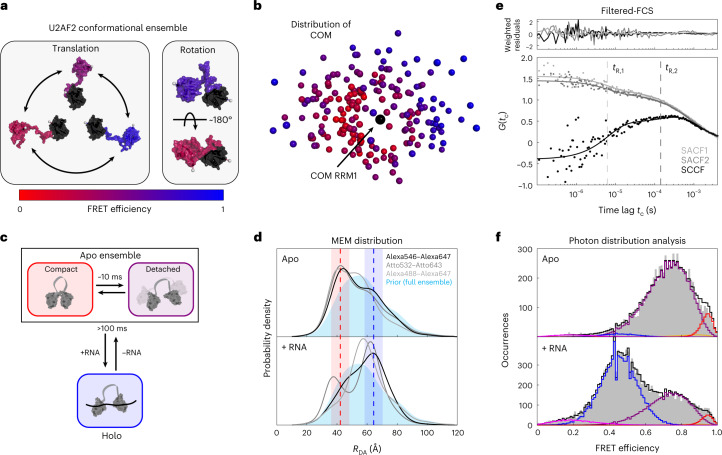
Structural characterization of U2AF2. **a**, Structural flexibility of U2AF2 is given by translational (left) and rotational (right) movement of the two domains. Representative structures are taken from the ensemble determined using NMR and SAXS measurements^29^. **b**, Degeneracy of structural states in FRET measurements. The position of the two domains of U2AF2 is illustrated by the COM of the C_α_ atoms in RRM2 (residues 260–329, colored) with respect to RRM1 (residues 150–227, black) for the 200 structures of the conformational ensemble^29^. The COM of RRM2 is color-coded according to the FRET efficiency determined using AV3 calculations. **c**, A schematic of the kinetic model used for the global dynamic PDA of U2AF2 (Supplementary Note 17). **d**, Distance distributions obtained from a donor fluorescence decay analysis by a model-free MEM approach (Supplementary Note 15). The distance distribution from the NMR–SAXS ensemble^29^ (light blue) was used as the prior distribution. The expected interdye distances for the compact apo and open holo states are shown as red and blue dashed lines (PDB 2YH0 and 2YH1). Shaded areas indicate the distance broadening due to the flexible dye linkers of 6 Å. The distribution in the donor–acceptor distance *R*_DA_ for different dye pairs is shown. **e**, Filtered-FCS reveals conformational dynamics in the U2AF2 apo ensemble on two timescales, *t*_*R*,1_ = 9 ± 3 and *t*_*R*,2_ = 300 ± 90 µs, average and standard deviation (*n* = 3, results from laboratory 1 are shown). The two species were defined at the lower and upper edge of the FRET efficiency histogram shown in Fig. 2b, top panel (see Methods and Supplementary Note 16 for details). The species autocorrelation functions (SACFs) and one of the two species cross-correlation functions (SCCFs) are shown. The weighted residuals are shown above. **f**, The PDA analysis was conducted globally over both apo (top) and holo (bottom) measurements using time windows of 0.5, 1.0, 1.5 and 2.0 ms (the 1.0 ms time window histograms are shown). A relaxation time of roughly 10 ms for the dynamics between the detached ensemble and compact apo state with a small amplitude was determined (orange curve) (Supplementary Fig. 16 and Supplementary Note 17). Source data

The structural characterization of U2AF2 is more complex than for MalE and a simple distance comparison is not possible. Nonetheless, we asked what information smFRET measurements could provide for such a dynamic system. We first surveyed the structural information available on apo U2AF2 from nuclear magnetic resonance (NMR) and small-angle X-ray scattering (SAXS) data^29^. The highly flexible linker allows for a heterogeneous ensemble of U2AF2 conformations (Fig. 6a). To assess how this translates into a smFRET distribution, we quantified the FRET efficiency using AV calculations for all 200 conformers from the NMR–SAXS-derived ensemble of apo U2AF2 (ref. ^29^). Notably, conformations with similar center-of-mass (COM) distances between the domains showed different FRET efficiencies (Fig. 6a,b), because domain rotations result in distinct interdye distances for identical COM (Fig. 6a, right). Due to this degeneracy, a single-distance probe is insufficient to capture the full structural complexity.

The observed ds in the apo state suggests the presence of conformational dynamics (Fig. 4d–f). To decipher the underlying kinetics and their temporal hierarchy, we applied three analyses. First, we investigated the interdye distance distribution of the apo and holo states from the donor lifetime using a model-free maximum entropy method (MEM) (Fig. 6c,d and Supplementary Note 15)^76^. As a prior, we used the NMR–SAXS structural ensemble. This analysis yielded consistent results for all three dye pairs studied for U2AF2. The MEM analysis revealed peaks in the probability density at the expected distances for the compact apo conformation and RNA-bound holo structure (Fig. 6d, dashed lines). We note that the fluorescence lifetime analysis resolves states on the nanosecond timescale and is therefore less sensitive to dynamic averaging.

Second, to assess the dynamics on the microsecond timescale, three groups performed filtered-FCS and found at least two relaxation times (9 ± 3 and 300 ± 90 µs; Fig. 6e, Supplementary Table 15 and Supplementary Note 16), which were independent of the dyes used (Supplementary Fig. 15). We assign the fast process to dynamics within the detached domains and the slower process to interconversion between compact conformations within the conformational ensemble.

Last, we investigated dynamics on the millisecond timescale using a dynamic PDA. A global analysis of the apo and holo measurements was performed using the kinetic model shown in Fig. 6c (Supplementary Note 17 and Supplementary Table 16). The apo state was treated as a two-state system with slow dynamics between a detached ensemble and a well-defined, compact apo conformation. The rapid dynamics within the detached ensemble is empirically described using a broad, static distribution. For the holo measurement, we account for the residual population of apo molecules. Exchange between the holo and apo states is irrelevant as the binding and dissociation of RNA occurs on timescales of more than 100 ms (ref. ^36^). This model incorporates all information and is sufficient to describe the smFRET efficiency histograms. The dynamic PDA analysis returned a relaxation time of roughly 10 ms for the dynamics between the detached ensemble and compact apo state (Fig. 6f, orange curve, Supplementary Fig. 16 and Supplementary Table 16). We also determined an interdye distance of *R*_〈*E*〉_ = 61 Å in the RNA-bound holo state, which is in good agreement with 63 Å from the RNA-bound conformation (Protein Data Bank (PDB) 2YH1).

## Discussion

We show that smFRET can provide accurate distances of conformational states and reliable information on conformational dynamics in proteins. Since all experiments were performed using established techniques and analyzed with freely available software^5,6,34,35,77–79^, such information is accessible to any group with similar expertise. Despite the challenges of protein samples, we achieved a similar precision in FRET efficiencies as reported for dsDNA^18^ (between ±0.02 and ±0.06) (Extended Data Table 1). The reproducibility in excluding large-scale conformational dynamics for MalE on a timescale <10 ms while detecting large-scale submillisecond dynamics in U2AF2 shows that the community can deal with dynamic protein systems. In addition, we could consistently establish the timescales and hierarchy of the exchange dynamics in such a complex protein system as U2AF2. The study of complex dynamics is improvable by probing additional distances^5,13,17,80–83^.

The high level of agreement is notable given the diversity of the setups (Fig. 3 and Supplementary Fig. 2) and the number of possible pitfalls. A large contribution to the spread in the reported mean FRET efficiencies was caused by systematic errors in the data analysis. This is supported by a comparison of the FRET efficiency changes ($\left\langle {E_{{{{\mathrm{holo}}}}}}\right\rangle - \left\langle{E_{{{{\mathrm{apo}}}}}} \right\rangle$) instead of absolute FRET efficiency values (Fig. 1d), which reduced the spread of roughly threefold. Having a single person reanalyze the data led to a similar decrease in the uncertainty of the FRET efficiency for the apo state of U2AF2 (Fig. 2d). Determination of *γ* was most crucial and the optimal approach depends on the details of the studied system (Supplementary Note 2). In the intensity-based approach of Lee et al.^34^, multiple samples with uniform fluorophore properties are required or individual corrections need to be made. When using the approach of Kudryavtsev et al.^35^ via *E*–*τ* calibration, the system needs to be static and a single population suffices. A protocol with unambiguous instructions for the calibration steps and minimized number of user-dependent steps would enhance the accuracy of FRET measurements.

From accurate FRET efficiencies, we obtained reproducible interdye distances with a precision of 3 Å and an accuracy of 5 Å against structural models of MalE (Extended Data Table 1). This is similar to what was determined for dsDNA samples. This is a very positive outcome, given that dsDNA features a consistent, homogenous chemical environment for each labeling position, in contrast to the variable dye environment experienced in proteins. The distance determination could be improved by including the interaction of the fluorophores with the protein surface using ACV calculations (Fig. 5d and Methods)^63^. Furthermore, we give experimental support (Fig. 5f) for only using dyes with a combined residual anisotropy of *r*_c,∞_ < 0.25, as suggested previously^13,70^. Proteins often exist within a family of conformations as we observed for U2AF2 (Fig. 6d). Determining how to best deal with distance distributions for conformational ensembles is one of the challenges for structural biology.

Investigating different dye pairs allowed us to reduce dye artifacts, leading to more accurate FRET efficiencies and reliable detection of the dynamics. Hence, we investigated the detection limits for ds and studied its relation to conformational dynamics with a subset of laboratories. Besides conformational motions, dynamic FRET shifts can occur in different directions and have several origins including structural instabilities^37^ or photophysics (as shown in Fig. 5f)^44^. Thus, it is advisable to verify the key findings in smFRET measurements with at least two dye pairs and/or with different residue combinations in the protein. Once the non-FRET-dynamic contributions are minimized, we still observed significant residual ds for MalE. Consistent with molecular dynamics simulations (Supplementary Note 13), we interpret these shifts as small-scale conformational dynamics and established a current lower limit for the detection of structural changes via smFRET on the order of ≤5 Å. In summary, the consensus of smFRET experiments on two protein systems exhibiting dynamic behavior on different spatiotemporal scales obtained blindly from 19 laboratories offers strong support for its use as a robust, versatile and quantitative tool for the coming age of dynamic structural biology. In this context, it will be crucial to integrate the correlated structural and dynamic information provided by smFRET^1^ with structural information provided by other experimental techniques as well as artificial intelligence-based protein structural prediction^83^. Considering that protein structure prediction has reached the single-structure frontier^84^, the information from smFRET experiments could leverage the power of artificial intelligence to resolve more complex multi-state and ensemble structural models^83^. Vice versa, the power of artificial intelligence and deep learning can be used to increase the throughput for the design and analysis of smFRET experiments^85–87^.

## Methods

### Sample preparation of proteins

Double-cysteine mutants of MalE were prepared and labeled using established protocols^62^. Human RRM1,2 L187C-G326C mutant (U2AF2-148-342) was obtained and purified as described in Mackereth et al.^30^.

### Fluorescence labeling of proteins

All fluorophores were purchased as maleimide derivatives from commercial suppliers as listed in Supplementary Table 19. MalE was stochastically labeled as described previously^88^ with fluorophores as indicated in the text with a combined labeling efficiency higher than 70% resulting in a donor–acceptor pairing of at least 20%. Protein stability and functionality (ligand binding) was verified by affinity measurements using microscale thermophoresis^89^. All preparations, that is, MalE-wildtype, unlabeled cysteine mutants and fluorophore-labeled variants, showed an affinity for maltose between roughly 1 and 2 µM (Supplementary Fig. 5) consistent with previously published *K*_d_values for wildtype MalE^90,91^. The stability and labeling of the sample were verified by FCS (Supplementary Fig. 18), which excluded the presence of larger aggregates in the samples and confirms that MalE is functional.

U2AF2 was stochastically labeled as described previously in Voith von Voithenberg et al.^36^. The combined labeling efficiencies for the labeling reactions were 20 and 14% for the Alexa546–Alexa647 and Atto532–Atto643 pairs, respectively. For Alexa488–Alexa647, the combined labeling efficiency was found to be 10%. The functionality of the labeled U2AF protein was checked with affinity measurements for U9 RNA, which was found to be 1.2 µM (ref. ^30^), consistent with the previous reports^36^ (Supplementary Fig. 7d).

### Sample handling

Both protein systems required special handling due to sample instability or aggregate formation, which are both problematic for long-term storage and shipping. The labeled MalE proteins were stored in 50 mM Tris-HCl pH 7.4, 50 mM KCl with 1 mg ml^−1^ BSA at 4 °C for less than 7 d. U2AF2 was stored in 20 mM potassium phosphate buffer pH 6.5, 50 mM NaCl and kept in the fridge until used. Both samples were loaded in low-binding Eppendorf tubes (Eppendorf Germany, catalog no. 0030108094) and shipped on ice in a cooling box with overnight shipping to avoid unnecessary freezing and thawing. MalE stock solutions were on the order of 10 to 100 nM concentration and the sent stock solution of U2AF2 was 5–10 µM concentration. Dilution buffers for apo and holo measurements were provided. SmFRET experiments were carried out by diluting the labeled proteins to concentrations of roughly 50 pM in 50 mM Tris-HCl pH 7.4, 50 mM KCl supplemented with the ligand maltose at 1 mM concentration. Labeled U2AF2 protein was measured at roughly 40–100 pM in 20 mM potassium phosphate buffer pH 6.5, 50 mM NaCl. Purchased U9 RNA (Biomers.net GmbH and IBA Solutions for Life Sciences) was dissolved in RNA-free water and added directly to the solution at a final concentration of 5 µM for the holo measurements. Both proteins were studied on coverslips typically passivated with 1 mg ml^−1^ BSA in buffer before adding the sample. The measurements were performed without any photostabilizer to keep the measurements as simple as possible to avoid any further source for discrepancies between the groups, for example, degradation of photostabilizer or use of different photostabilizer concentrations.

### SmFRET data acquisition and analysis

Data acquisition and correction procedures were performed for confocal measurements as described by Hellenkamp et al.^18^. The samples were measured using ALEX or PIE on a confocal microscope as sketched in Supplementary Fig. 2. A description of the experimental procedures of all laboratories is given in Supplementary Note 18.

Briefly, the three recorded intensity time traces for each single-molecule event are:

$${{{\mathrm{donor}}}}\,{{{\mathrm{emission}}}}\,{{{\mathrm{after}}}}\,{{{\mathrm{donor}}}}\,{{{\mathrm{excitation}}}}:{}^{{{\mathrm{i}}}}I_{{{{\mathrm{Dem}}}}|{{{\mathrm{Dex}}}}},$$

$${{{\mathrm{acceptor}}}}\,{{{\mathrm{emission}}}}\,{{{\mathrm{after}}}}\,{{{\mathrm{donor}}}}\,{{{\mathrm{excitation}}}}\left( {{{{\mathrm{FRET}}}}\,{{{\mathrm{signal}}}}} \right):{}^{{{\mathrm{i}}}}I_{{{{\mathrm{Aem}}}}|{{{\mathrm{Dex}}}}},$$

$${{{\mathrm{and}}}}\,{{{\mathrm{acceptor}}}}\,{{{\mathrm{emission}}}}\,{{{\mathrm{after}}}}\,{{{\mathrm{acceptor}}}}\,{{{\mathrm{excitation}}}}:{}^{{{\mathrm{i}}}}I_{{{{\mathrm{Aem}}}}|{{{\mathrm{Dex}}}}}.$$

The apparent (raw) FRET efficiency is computed as:

3 $$E_{{{{\mathrm{app}}}}} = \frac{{{}^{{{\mathrm{i}}}}I_{{{{\mathrm{Aem}}}}|{{{\mathrm{Dex}}}}}}}{{{}^{{{\mathrm{i}}}}I_{{{{\mathrm{Dem}}}}|{{{\mathrm{Dex}}}}} + {}^{{{\mathrm{i}}}}I_{{{{\mathrm{Aem}}}}|{{{\mathrm{Dex}}}}}}},$$

Recorded intensities were corrected for background contributions as:

4 $${}^{{{{\mathrm{ii}}}}}I_{{{{\mathrm{Dem}}}}|{{{\mathrm{Dex}}}}} = {}^{{{\mathrm{i}}}}I_{{{{\mathrm{Dem}}}}|{{{\mathrm{Dex}}}}} - {}^{{{\mathrm{i}}}}I_{{{{\mathrm{Dem}}}}|{{{\mathrm{Dex}}}}}^{\,({{{\mathrm{BG}}}})},$$

5 $${}^{{{{\mathrm{ii}}}}}I_{{{{\mathrm{Aem}}}}|{{{\mathrm{Dex}}}}} = {}^{{{\mathrm{i}}}}I_{{{{\mathrm{Aem}}}}|{{{\mathrm{Dex}}}}} - I_{{{{\mathrm{Aem}}}}|{{{\mathrm{Dex}}}}}^{\,({{{\mathrm{BG}}}})},$$

6 $${}^{{{{\mathrm{ii}}}}}I_{{{{\mathrm{Aem}}}}|{{{\mathrm{Aex}}}}} = {}^{{{\mathrm{i}}}}I_{{{{\mathrm{Aem}}}}|{{{\mathrm{Aex}}}}} - I_{{{{\mathrm{Aem}}}}|{{{\mathrm{Aex}}}}}^{\,({{{\mathrm{BG}}}})},$$

where $I_{{{{\mathrm{Dem}}}}|{{{\mathrm{Dex}}}}}^{\,({{{\mathrm{BG}}}})}$, $I_{{{{\mathrm{Aem}}}}|{{{\mathrm{Dex}}}}}^{\,({{{\mathrm{BG}}}})}$ and $I_{{{{\mathrm{Aem}}}}|{{{\mathrm{Aex}}}}}^{\,({{{\mathrm{BG}}}})}$ are the respective background signals. Correction factors for spectral crosstalk, *α* and direct excitation, *δ*, were determined from the donor- and acceptor-only populations^34^. The corrected acceptor fluorescence after donor excitation, $F_{{\mathrm{A|D}}}$, is computed as:

7 $$F_{{\mathrm{A|D}}} = {}^{{{{\mathrm{ii}}}}}I_{{{{\mathrm{Aem}}}}|{{{\mathrm{Dex}}}}} - \alpha \;{}^{{{{\mathrm{ii}}}}}I_{{{{\mathrm{Dem}}}}|{{{\mathrm{Dex}}}}} - \delta \;{}^{{{{\mathrm{ii}}}}}I_{{{{\mathrm{Aem}}}}|{{{\mathrm{Aex}}}}}$$

The *γ* and *β* factors, correcting for differences in the detection yield and excitation fluxes of the donor and acceptor dyes, were estimated using a global correction procedure following the approach of Lee et al. (Supplementary Fig. 3)^34^. Alternatively, when pulsed excitation was used and the sample is known to be static, the *γ* factor can be determined by fitting the measured population to the static FRET line^35,92^. This allows a robust determination of the *γ* factor when only a single species is present but requires a static sample and the appropriate static FRET line (Supplementary Note 2).

The accurate FRET efficiency *E* and stoichiometry *S* values were then calculated as:

8 $$E = \frac{{F_{{\mathrm{A|D}}}}}{{\gamma \;{}^{{{{\mathrm{ii}}}}}I_{{{{\mathrm{Dem}}}}|{{{\mathrm{Dex}}}}} + F_{{\mathrm{A|D}}}}},$$

9 $$S = \frac{{\gamma \; {}^{{{{\mathrm{ii}}}}}I_{{{{\mathrm{Dem}}}}|{{{\mathrm{Dex}}}}} + F_{{\mathrm{A|D}}}}}{{\gamma \;{}^{{{{\mathrm{ii}}}}}I_{{{{\mathrm{Dem}}}}|{{{\mathrm{Dex}}}}} + F_{{\mathrm{A|D}}} + {}^{{{{\mathrm{ii}}}}}I_{{{{\mathrm{Aem}}}}|{{{\mathrm{Aex}}}}}/\beta }}.$$

Conversion of accurate FRET efficiencies into distances were done using equation (2) with Förster radii determined as described in Supplementary Note 7.

### Detection of protein dynamics

In this work, we used the following two approaches to detect conformational dynamics:

#### BVA

In BVA, the presence of dynamics is determined by looking for excess variance in the FRET efficiency data beyond the shot-noise limit. The standard deviation ($\sigma _{E_{{{{\mathrm{app}}}}}}$) of the apparent FRET efficiency (*E*_app_) is calculated using a fixed photon window of *n* = 5 over the time period of the individual bursts given by:

10 $$\begin{array}{*{20}{c}} {\sigma _{E_{{{{\mathrm{app}}}}}} = \sqrt {\frac{{E_{{{{\mathrm{app}}}}}\left( {1 - E_{{{{\mathrm{app}}}}}} \right)}}{n}} ,} \end{array}$$

The shot-noise limited standard deviation of the apparent FRET efficiency is generally described by a semicircle^50^ (Fig. 4a and Supplementary Fig. 11a–d). In the presence of dynamics, the standard deviation for the FRET efficiency within a burst becomes higher than that expected from shot noise. Photophysical effects such as photobleaching and blinking also give rise to the higher standard deviation beyond the shot-noise limit. Typically, BVA is sensitive to fluctuations in the FRET signal of ≳100 µs, but this depends on the brightness of the burst and the photon window used.

#### FRET efficiency versus fluorescence-weighted average donor lifetime analysis (*E–*τ plots)

Two-dimensional histograms of the FRET efficiency *E* and donor fluorescence lifetime $\left\langle {\tau _{{\mathrm{D}}\left( {\mathrm{A}} \right)}} \right\rangle _{\mathrm{F}}$ (Fig. 4b and Supplementary Fig. 11e–h) were created for single-molecule measurements using multiparameter fluorescence detection (MFD) in combination with PIE^35^, described below. Static FRET lines were calculated using the following equation:

11 $$E = 1 - \frac{{\tau _{{\mathrm{D}}({\mathrm{A}})}}}{{\tau _{{\mathrm{D}}(0)}}}$$

and further modified for linker dynamics^61^. Deviations of FRET populations from the static FRET line can indicate FRET dynamics, which can be due to conformational fluctuations or photophysical dynamics. In addition, a time-resolved FRET analysis of TCSPC data can accurately resolve the distance heterogeneities by revealing multiple components in the decay curve and recovering their specific species fractions and FRET rate constants^69^. Dynamics are thus detected from the presence of multiple components in the subensemble decay of a single FRET population. In addition, dynamics that are slower than the fluorescence lifetime (roughly 5 ns) are not averaged in the FRET lifetime analysis leading to the detection of the full conformational distribution.

### MFD with PIE

MFD, introduced by Eggeling et al.^93^, combines spectral and polarized detection with picosecond pulsed lasers and TCSPC, allowing the simultaneous detection of intensity, lifetime, anisotropy and spectral range of the fluorescence signal of single molecules. nsALEX or PIE additionally provides the acceptor lifetime information^35^. Due to the availability of the lifetime information when using pulsed excitation, this approach is well suited for using *E*–τ-based analyses.

### Dye simulations (AV and ACV)

The AV approach uses a simple coarse-grained dye model^64^ defined by five parameters: the width and length of the linker, and three radii that define the fluorophore volume (Fig. 5a and Supplementary Table 10). Using these parameters, AV simulations for both fluorophores were calculated by considering the linker flexibility and steric hindrances of the labeled molecule (Fig. 5a). In the ACV model^63^, the position of the dyes is biased toward the protein surface, resulting in a reduction of the interdye distance for the given labeling positions. To do this, the residual anisotropy was used to estimate the fraction of sticking dyes. In the computation of the FRET-averaged model distances, the occupancy of a thin surface layer (roughly 3 Å) was then increased such that its fraction matches the amount of interacting dye detected in the experiment (Fig. 5b and Supplementary Table 10).

### Reporting summary

Further information on research design is available in the Nature Portfolio Reporting Summary linked to this article.

## Online content

Any methods, additional references, Nature Portfolio reporting summaries, source data, extended data, supplementary information, acknowledgements, peer review information; details of author contributions and competing interests; and statements of data and code availability are available at 10.1038/s41592-023-01807-0.

### Supplementary information

Supplementary Information Supplementary Notes 1–18, Figs. 1–19, Tables 1–19 and References.

Reporting Summary

Peer reviewer file

### Source data

Source Data Fig. 1 Excel sheet.

Source Data Fig. 2 Excel sheet.

Source Data Fig. 3 Excel sheet.

Source Data Fig. 4 Excel sheet.

Source Data Fig. 5 Excel sheet.

Source Data Fig. 6 Excel sheet.

## Figures and Tables

**Extended Data Table. 1:**
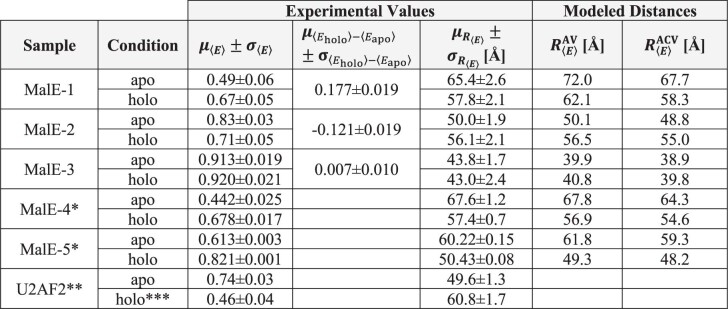
Average of mean FRET efficiency and standard deviation for MalE and U2AF2 samples reported by the participating laboratories

The calculated average $\mu _{\left\langle E \right\rangle }$ and standard deviation $\sigma _{\left\langle E \right\rangle }$ of the mean FRET efficiency values provided by the participating labs are given for all three studied mutants of MalE labeled with Alexa546 and Alexa647 under both apo and holo conditions (see Supplementary Table 3). The calculated mean and standard deviation of the difference in the reported mean FRET efficiency between the apo and holo ($\left\langle {E_{{{{\mathrm{holo}}}}}}\right\rangle - \left\langle{E_{{{{\mathrm{apo}}}}}} \right\rangle$) for the three MalE mutants are given by$\mu _{\left\langle {E_{{{{\mathrm{holo}}}}}}\right\rangle - \left\langle{E_{{{{\mathrm{apo}}}}}} \right\rangle }$ and ${{{\mathrm{\sigma }}}}_{\left\langle {E_{{{{\mathrm{holo}}}}}}\right\rangle - \left\langle{E_{{{{\mathrm{apo}}}}}} \right\rangle }$ respectively (see Supplementary Table 3). The calculated average $\mu _{R_{\left\langle E \right\rangle }}$ and standard deviation $\sigma _{R_{\left\langle E \right\rangle }}$ of the mean distances were derived according to Eq. 2. The modeled distances $R_{\left\langle E \right\rangle }^{{{{\mathrm{AV}}}}}$ and $R_{\left\langle E \right\rangle }^{{{{\mathrm{ACV}}}}}$ are derived using accessible volume (AV) and accessible contact volume (ACV) calculations respectively, as described in the Methods. We also give the average and standard deviation for the FRET values determined for U2AF2 labeled with Atto532-Atto643 under both apo and holo conditions (Supplementary Table 4).

*Only studied by two laboratories.

**Due to the fast-structural dynamics in the sample, only 7 labs studied this mutant and distances were not determined.

*** Only the holo state under holo condition was considered.

## Data Availability

The data for all figures, all supplementary figures, the raw data for MalE measurements from all laboratories (with the exception of one mutant from one laboratory) and the raw data for all U2AF2 measurements have been uploaded to Zenodo (10.5281/zenodo.7472900). PDB IDs used are 1OMP, 1ANF, 2YHO and 2YH1. Source data are provided with this paper.

## Code availability

The software used for data analysis are available from the respective laboratories: laboratory no. 1, PAM (PIE Analysis with MATLAB) software package (ref. ^79^ in the main text); laboratory no. 2, Home-written LabView-based software (ref. ^15^ in Supplementary Information); Laboratory no. 3, FRETBursts toolkit (ref. ^14^ in Supplementary Information); laboratory no. 4, PAM (PIE Analysis with MATLAB) software package; laboratory no. 5, PAM (PIE Analysis with MATLAB) software package; laboratory no. 6, PAM (PIE Analysis with MATLAB) software package; laboratory no. 7, PAM (PIE Analysis with MATLAB) software package; laboratory no. 8, PAM (PIE Analysis with MATLAB) software package v.2.0; laboratory no. 9, PAM (PIE Analysis with MATLAB) software package; laboratory no. 11, data were analyzed with the burst analysis toolbox (BAT, V2018 and V2019) and filtered and visualized with T3ee (V2018, V2019) (ref. ^84^ in Supplementary Information); laboratory no. 12: PAM (PIE Analysis with MATLAB) software package; laboratory no. 13, IgorPro 8 (Wavemetrics); laboratory no. 14, PAM (PIE Analysis with MATLAB) software package; laboratory no. 15, Software Package for Multiparameter Fluorescence Spectroscopy, Full Correlation and Multiparameter Fluorescence Imaging developed in C.A.M. Seidel’s laboratory (http://www.mpc.uni-duesseldorf.de/seidel/); laboratory no. 16, ALEX-suite software package (ref. ^88^ of Supplementary Information); laboratory no. 17, FRETBursts analysis software (ref. ^14^ of Supplementary Information) and laboratory no. 18, FRETBursts toolkit (ref. ^14^ of Supplementary Information).

## References

[1] Lerner E (2021). FRET-based dynamic structural biology: challenges, perspectives and an appeal for open-science practices. eLife.

[2] Lerner E (2018). Toward dynamic structural biology: two decades of single-molecule Förster resonance energy transfer. Science.

[3] Algar WR, Hildebrandt N, Vogel SS, Medintz IL (2019). FRET as a biomolecular research tool—understanding its potential while avoiding pitfalls. Nat. Methods.

[4] Hildebrandt, N. in *FRET—**Förster Resonance Energy Transfer* (eds Medintz, I. & Hildebrandt, N.) 105–163 (Wiley, 2013).

[5] Muschielok A (2008). A nano-positioning system for macromolecular structural analysis. Nat. Methods.

[6] Kalinin S (2012). A toolkit and benchmark study for FRET-restrained high-precision structural modeling. Nat. Methods.

[7] Craggs TD, Kapanidis AN (2012). Six steps closer to FRET-driven structural biology. Nat. Methods.

[8] Voith von Voithenberg L, Lamb DC (2018). Single pair Förster resonance energy transfer: a versatile tool to investigate protein conformational dynamics. BioEssays.

[9] Hohlbein J, Craggs TD, Cordes T (2014). Alternating-laser excitation: single-molecule FRET and beyond. Chem. Soc. Rev..

[10] Krainer G, Hartmann A, Schlierf M (2015). FarFRET: extending the range in single-molecule FRET experiments beyond 10 nm. Nano Lett..

[11] Muschielok A, Michaelis J (2011). Application of the nano-positioning system to the analysis of fluorescence resonance energy transfer networks. J. Phys. Chem. B..

[12] Sali A (2015). Outcome of the first wwPDB Hybrid/Integrative Methods Task Force Workshop. Structure.

[13] Hellenkamp B, Wortmann P, Kandzia F, Zacharias M, Hugel T (2017). Multidomain structure and correlated dynamics determined by self-consistent FRET networks. Nat. Methods.

[14] Choi UB (2010). Single-molecule FRET-derived model of the synaptotagmin 1-SNARE fusion complex. Nat. Struct. Mol. Biol..

[15] Dimura M (2020). Automated and optimally FRET-assisted structural modeling. Nat. Commun..

[16] Lerner E, Ingargiola A, Weiss S (2018). Characterizing highly dynamic conformational states: the transcription bubble in RNAP-promoter open complex as an example. J. Chem. Phys..

[17] Craggs TD (2019). Substrate conformational dynamics facilitate structure-specific recognition of gapped DNA by DNA polymerase. Nucleic Acids Res..

[18] Hellenkamp B (2018). Precision and accuracy of single-molecule FRET measurements—a multi-laboratory benchmark study. Nat. Methods.

[19] Rout MP, Sali A (2019). Principles for integrative structural biology studies. Cell.

[20] Sali A (2021). From integrative structural biology to cell biology. J. Biol. Chem..

[21] Burley SK (2017). PDB-Dev: a prototype system for depositing integrative/hybrid structural models. Structure.

[22] Davidson AL, Dassa E, Orelle C, Chen J (2008). Structure, function, and evolution of bacterial ATP-binding cassette systems. Microbiol. Mol. Biol. Rev..

[23] Mächtel R, Narducci A, Griffith DA, Cordes T, Orelle C (2019). An integrated transport mechanism of the maltose ABC importer. Res. Microbiol..

[24] Malik A (2016). Protein fusion tags for efficient expression and purification of recombinant proteins in the periplasmic space of *E. coli*. 3 Biotech.

[25] Berntsson RPA, Smits SHJ, Schmitt L, Slotboom DJ, Poolman B (2010). A structural classification of substrate-binding proteins. FEBS Lett..

[26] Fukami-Kobayashi K, Tateno Y, Nishikawa K (1999). Domain dislocation: a change of core structure in periplasmic binding proteins in their evolutionary history. J. Mol. Biol..

[27] Banerjee H, Rahn A, Davis W, Singh R (2003). Sex lethal and U2 small nuclear ribonucleoprotein auxiliary factor (U2AF65) recognize polypyrimidine tracts using multiple modes of binding. RNA.

[28] Sickmier EA (2006). Structural basis for polypyrimidine tract recognition by the essential pre-mRNA splicing factor U2AF65. Mol. Cell.

[29] Huang JR (2014). Transient electrostatic interactions dominate the conformational equilibrium sampled by multidomain splicing factor U2AF65: a combined NMR and SAXS study. J. Am. Chem. Soc..

[30] MacKereth CD (2011). Multi-domain conformational selection underlies pre-mRNA splicing regulation by U2AF. Nature.

[31] Kapanidis AN (2004). Fluorescence-aided molecule sorting: analysis of structure and interactions by alternating-laser excitation of single molecules. Proc. Natl Acad. Sci. USA.

[32] Kapanidis AN (2005). Alternating-laser excitation of single molecules. Acc. Chem. Res..

[33] Müller BK, Zaychikov E, Bräuchle C, Lamb DC (2005). Pulsed interleaved excitation. Biophys. J..

[34] Lee NK (2005). Accurate FRET measurements within single diffusing biomolecules using alternating-laser excitation. Biophys. J..

[35] Kudryavtsev V (2012). Combining MFD and PIE for accurate single-pair Förster resonance energy transfer measurements. Chem. Phys. Chem..

[36] Von Voithenberg LV (2016). Recognition of the 3′ splice site RNA by the U2AF heterodimer involves a dynamic population shift. Proc. Natl Acad. Sci. USA.

[37] Sánchez-Rico C, Voith von Voithenberg L, Warner L, Lamb DC, Sattler M (2017). Effects of fluorophore attachment on protein conformation and dynamics studied by spFRET and NMR spectroscopy. Chemistry.

[38] Eggeling C, Widengren J, Rigler R, Seidel CAM (1998). Photobleaching of fluorescent dyes under conditions used for single-molecule detection: evidence of two-step photolysis. Anal. Chem..

[39] Chung HS, McHale K, Louis JM, Eaton WA (2012). Single-molecule fluorescence experiments determine protein folding transition path times. Science.

[40] Ramanathan R, Muñoz V (2015). A method for extracting the free energy surface and conformational dynamics of fast-folding proteins from single molecule photon trajectories. J. Phys. Chem. B..

[41] McKinney SA, Joo C, Ha T (2006). Analysis of single-molecule FRET trajectories using hidden Markov modeling. Biophys. J..

[42] Liu Y, Park J, Dahmen KA, Chemla YR, Ha T (2010). A comparative study of multivariate and univariate hidden Markov modelings in time-binned single-molecule FRET data analysis. J. Phys. Chem. B..

[43] Bronson JE, Fei J, Hofman JM, Gonzalez RL, Wiggins CH (2009). Learning rates and states from biophysical time series: a Bayesian approach to model selection and single-molecule FRET data. Biophys. J..

[44] Margittai M (2003). Single-molecule fluorescence resonance energy transfer reveals a dynamic equilibrium between closed and open conformations of syntaxin 1. Proc. Natl Acad. Sci. USA.

[45] Diez M (2004). Proton-powered subunit rotation in single membrane-bound F 0F1-ATP synthase. Nat. Struct. Mol. Biol..

[46] Torres T, Levitus M (2007). Measuring conformational dynamics: a new FCS-FRET approach. J. Phys. Chem. B..

[47] Felekyan, S., Sanabria, H., Kalinin, S., Kühnemuth, R. & Seidel, C. A. M. Analyzing Förster resonance energy transfer with fluctuation algorithms. *Methods Enzymol.* **519**, 39–85 (2013). 10.1016/B978-0-12-405539-1.00002-6 23280107

[48] Felekyan S, Kalinin S, Sanabria H, Valeri A, Seidel CAM (2012). Filtered FCS: species auto- and cross-correlation functions highlight binding and dynamics in biomolecules. Chem. Phys. Chem..

[49] Olofsson L (2014). Fine tuning of sub-millisecond conformational dynamics controls metabotropic glutamate receptors agonist efficacy. Nat. Commun..

[50] Torella JP, Holden SJ, Santoso Y, Hohlbein J, Kapanidis AN (2011). Identifying molecular dynamics in single-molecule FRET experiments with burst variance analysis. Biophys. J..

[51] Tomov TE (2012). Disentangling subpopulations in single-molecule FRET and ALEX experiments with photon distribution analysis. Biophys. J..

[52] Kalinin S, Valeri A, Antonik M, Felekyan S, Seidel CAM (2010). Detection of structural dynamics by FRET: a photon distribution and fluorescence lifetime analysis of systems with multiple states. J. Phys. Chem. B..

[53] Gopich IV, Szabo A (2012). Theory of the energy transfer efficiency and fluorescence lifetime distribution in single-molecule FRET. Proc. Natl Acad. Sci. USA.

[54] Nettels D, Gopich IV, Hoffmann A, Schuler B (2007). Ultrafast dynamics of protein collapse from single-molecule photon statistics. Proc. Natl Acad. Sci. USA.

[55] Hoffmann A (2011). Quantifying heterogeneity and conformational dynamics from single molecule FRET of diffusing molecules: recurrence analysis of single particles (RASP). Phys. Chem. Chem. Phys..

[56] Gopich IV, Szabo A (2009). Decoding the pattern of photon colors in single-molecule FRET. J. Phys. Chem. B..

[57] Chung HS, Gopich IV (2014). Fast single-molecule FRET spectroscopy: theory and experiment. Phys. Chem. Chem. Phys..

[58] Pirchi M (2016). Photon-by-photon hidden Markov model analysis for microsecond single-molecule FRET kinetics. J. Phys. Chem. B..

[59] Harris PD (2022). Multi-parameter photon-by-photon hidden Markov modeling. Nat. Commun..

[60] Ingargiola A, Weiss S, Lerner E (2018). Monte Carlo diffusion-enhanced photon inference: distance distributions and conformational dynamics in single-molecule FRET. J. Phys. Chem. B..

[61] Barth A (2022). Unraveling multi-state molecular dynamics in single-molecule FRET experiments. I. Theory of FRET-lines. J. Chem. Phys..

[62] De Boer M (2019). Conformational and dynamic plasticity in substrate-binding proteins underlies selective transport in ABC importers. eLife.

[63] Dimura M (2016). Quantitative FRET studies and integrative modeling unravel the structure and dynamics of biomolecular systems. Curr. Opin. Struct. Biol..

[64] Sindbert S (2011). Accurate distance determination of nucleic acids via Förster resonance energy transfer: implications of dye Linker length and rigidity. J. Am. Chem. Soc..

[65] Steffen FD, Sigel RKO, Börner R (2016). An atomistic view on carbocyanine photophysics in the realm of RNA. Phys. Chem. Chem. Phys..

[66] Klose D (2021). Resolving distance variations by single-molecule FRET and EPR spectroscopy using rotamer libraries. Biophys. J..

[67] Reinartz I (2018). Simulation of FRET dyes allows quantitative comparison against experimental data. J. Chem. Phys..

[68] Hoefling M (2011). Structural heterogeneity and quantitative FRET efficiency distributions of polyprolines through a hybrid atomistic simulation and monte carlo approach. PLoS ONE.

[69] Peulen TO, Opanasyuk O, Seidel CAM (2017). Combining graphical and analytical methods with molecular simulations to analyze time-resolved FRET measurements of labeled macromolecules accurately. J. Phys. Chem. B..

[70] Dale RE, Eisinger J, Blumberg WE (1979). The orientational freedom of molecular probes. The orientation factor in intramolecular energy transfer. Biophys. J..

[71] Dale RE, Eisinger J (1974). Intramolecular distances determined by energy transfer. Dependence on orientational freedom of donor and acceptor. Biopolymers.

[72] Ivanov V, Li M, Mizuuchi K (2009). Impact of emission anisotropy on fluorescence spectroscopy and FRET distance measurements. Biophys. J..

[73] Eilert T, Kallis E, Nagy J, Röcker C, Michaelis J (2018). Complete kinetic theory of FRET. J. Phys. Chem. B.

[74] Maier JA (2015). ff14SB: improving the accuracy of protein side chain and backbone parameters from ff99SB. J. Chem. Theory Comput..

[75] Zaccai G (2000). How soft is a protein? A protein dynamics force constant measured by neutron scattering. Science.

[76] Vinogradov SA, Wilson DF (2000). Recursive maximum entropy algorithm and its application to the luminescence lifetime distribution recovery. Appl. Spectrosc..

[77] Ingargiola A, Lerner E, Chung SY, Weiss S, Michalet X (2016). FRETBursts: an open source toolkit for analysis of freely-diffusing Single-molecule FRET. PLoS ONE.

[78] Schrimpf W, Barth A, Hendrix J, Lamb DC (2018). PAM: a framework for integrated analysis of imaging, single-molecule, and ensemble fluorescence data. Biophys. J..

[79] Ambrose B (2020). The smfBox is an open-source platform for single-molecule FRET. Nat. Commun..

[80] Knight JL, Mekler V, Mukhopadhyay J, Ebright RH, Levy RM (2005). Distance-restrained docking of rifampicin and rifamycin SV to RNA polymerase using systematic FRET measurements: developing benchmarks of model quality and reliability. Biophys. J..

[81] Kapanidis AN (2006). Initial transcription by RNA polymerase proceeds through a DNA-scrunching mechanism. Science.

[82] Sanabria H (2020). Resolving dynamics and function of transient states in single enzyme molecules. Nat. Commun..

[83] Berman HM (2019). Federating structural models and data: outcomes from a workshop on archiving integrative. Structures. Structure.

[84] Lane, T. J. Protein structure prediction has reached the single-structure frontier. *Nat. Methods* **20**, 170–173 (2023). 10.1038/s41592-022-01760-4 PMC9839224 36639584

[85] Li J, Zhang L, Johnson-Buck A, Walter NG (2020). Automatic classification and segmentation of single-molecule fluorescence time traces with deep learning. Nat. Commun..

[86] Thomsen J (2020). DeepFRET, a software for rapid and automated single-molecule FRET data classification using deep learning. eLife.

[87] Wanninger, S. et al. Deep-learning assisted, single-molecule imaging analysis (Deep-LASI) of multi-color DNA Origami structures. Preprint at *bioRxiv*10.1101/2023.01.31.526220 (2023). 10.1038/s41467-023-42272-9 PMC10582187 37848439

[88] Gouridis G (2015). Conformational dynamics in substrate-binding domains influences transport in the ABC importer GlnPQ. Nat. Struct. Mol. Biol..

[89] Jerabek-Willemsen M (2014). MicroScale thermophoresis: interaction analysis and beyond. J. Mol. Struct..

[90] Hall JA, Gehring K, Nikaido H (1997). Two modes of ligand binding in maltose-binding protein of *Escherichia coli*: correlation with the structure of ligands and the structure of binding protein. J. Biol. Chem..

[91] Kim E (2013). A single-molecule dissection of ligand binding to a protein with intrinsic dynamics. Nat. Chem. Biol..

[92] Sisamakis E, Valeri A, Kalinin S, Rothwell PJ, Seidel CAM (2010). Accurate single-molecule FRET studies using multiparameter fluorescence detection. Methods Enzymol..

[93] Eggeling C (2001). Data registration and selective single-molecule analysis using multi-parameter fluorescence detection. J. Biotechnol..

