# Supplementary material for: Reliability and accuracy of single-molecule FRET studies for characterization of structural dynamics and distances in proteins
Source: Nat Methods. 2023 Mar 27;20(4):523–35. doi: 10.1038/s41592-023-01807-0 (PMC10089922; doi:10.1038/s41592-023-01807-0)
Supplement: Supplementary file 3 — Peer reviewer file [file 41592_2023_1807_MOESM3_ESM.pdf]

natureresearch

Peer Review Information

Journal: Nature Methods
Manuscript Title: Reliability and accuracy of single-molecule FRET studies for characterization of structural dynamics and distances in proteins
Corresponding author name: Don Lamb

Editorial Notes: n/a

Reviewer Comments & Decisions:

Decision Letter, initial version:

Dear Don,

Your Analysis, "Reliability and accuracy of single-molecule FRET studies for characterization of structural dynamics and distances in proteins", has now been seen by two reviewers. As you will see from their comments below, although the reviewers find your work of considerable potential interest, they have raised some concerns. We are interested in the possibility of publishing your paper in Nature Methods, but would like to consider your response to these concerns before we reach a final decision on publication.

We therefore invite you to revise your manuscript to address these concerns, which largely involve clarification of how the study was conducted, but potentially include some new analyses. We do not ask that experiments that were not conducted by all labs be done by more labs, we simply ask you to clarify how many groups conducted each set of experiments. This was a point of confusion.

We are committed to providing a fair and constructive peer-review process. Do not hesitate to contact us if there are specific requests from the reviewers that you believe are technically impossible or unlikely to yield a meaningful outcome.

When revising your paper:

* include a point-by-point response to the reviewers and to any editorial suggestions

* please underline/highlight any additions to the text or areas with other significant changes to facilitate review of the revised manuscript

* address the points listed described below to conform to our open science requirements

* ensure it complies with our general format requirements as set out in our guide to authors at www.nature.com/naturemethods

* resubmit all the necessary files electronically by using the link below to access your home page

[Redacted] This URL links to your confidential home page and associated information about manuscripts you may have submitted, or that you are reviewing for us. If you wish to forward this email to co-authors, please delete the link to your homepage.

We hope to receive your revised paper within two months. If you cannot send it within this time, please let us know. In this event, we will still be happy to reconsider your paper at a later date so long as nothing similar has been accepted for publication at Nature Methods or published elsewhere.

OPEN SCIENCE REQUIREMENTS

REPORTING SUMMARY AND EDITORIAL POLICY CHECKLISTS
When revising your manuscript, please update your reporting summary and editorial policy checklists.

Reporting summary: https://www.nature.com/documents/nr-reporting-summary.zip
Editorial policy checklist: https://www.nature.com/documents/nr-editorial-policy-checklist.zip

If your paper includes custom software, we also ask you to complete a supplemental reporting summary.

Software supplement: https://www.nature.com/documents/nr-software-policy.pdf

Please submit these with your revised manuscript. They will be available to reviewers to aid in their evaluation if the paper is re-reviewed. If you have any questions about the checklist, please see http://www.nature.com/authors/policies/availability.html or contact me.

Please note that these forms are dynamic ‘smart pdfs’ and must therefore be downloaded and completed in Adobe Reader. We will then flatten them for ease of use by the reviewers. If you would like to reference the guidance text as you complete the template, please access these flattened versions at http://www.nature.com/authors/policies/availability.html.

IMAGE INTEGRITY
When submitting the revised version of your manuscript, please pay close attention to our Digital Image Integrity Guidelines and to the following points below:

-- that unprocessed scans are clearly labelled and match the gels and western blots presented in figures.
-- that control panels for gels and western blots are appropriately described as loading on sample processing controls
-- all images in the paper are checked for duplication of panels and for splicing of gel lanes.

Finally, please ensure that you retain unprocessed data and metadata files after publication, ideally archiving data in perpetuity, as these may be requested during the peer review and production process or after publication if any issues arise.

DATA AVAILABILITY
We strongly encourage you to deposit all new data associated with the paper in a persistent repository where they can be freely and enduringly accessed. We recommend submitting the data to discipline-specific and community-recognized repositories; a list of repositories is provided here: http://www.nature.com/sdata/policies/repositories

All novel DNA and RNA sequencing data, protein sequences, genetic polymorphisms, linked genotype and phenotype data, gene expression data, macromolecular structures, and proteomics data must be deposited in a publicly accessible database, and accession codes and associated hyperlinks must be provided in the “Data Availability” section.

Refer to our data policies here: https://www.nature.com/nature-research/editorial-policies/reporting-standards#availability-of-data

To further increase transparency, we encourage you to provide, in tabular form, the data underlying the graphical representations used in your figures. This is in addition to our data-deposition policy for specific types of experiments and large datasets. For readers, the source data will be made accessible

directly from the figure legend. Spreadsheets can be submitted in .xls, .xlsx or .csv formats. Only one (1) file per figure is permitted: thus if there is a multi-paneled figure the source data for each panel should be clearly labeled in the csv/Excel file; alternately the data for a figure can be included in multiple, clearly labeled sheets in an Excel file. File sizes of up to 30 MB are permitted. When submitting source data files with your manuscript please select the Source Data file type and use the Title field in the File Description tab to indicate which figure the source data pertains to.

Please include a “Data availability” subsection in the Online Methods. This section should inform readers about the availability of the data used to support the conclusions of your study, including accession codes to public repositories, references to source data that may be published alongside the paper, unique identifiers such as URLs to data repository entries, or data set DOIs, and any other statement about data availability. At a minimum, you should include the following statement: “The data that support the findings of this study are available from the corresponding author upon request”, describing which data is available upon request and mentioning any restrictions on availability. If DOIs are provided, please include these in the Reference list (authors, title, publisher (repository name), identifier, year). For more guidance on how to write this section please see:
http://www.nature.com/authors/policies/data/data-availability-statements-data-citations.pdf

CODE AVAILABILITY
Please include a “Code Availability” subsection in the Online Methods which details how your custom code is made available. Only in rare cases (where code is not central to the main conclusions of the paper) is the statement “available upon request” allowed (and reasons should be specified).

We request that you deposit code in a DOI-minting repository such as Zenodo, Gigantum or Code Ocean and cite the DOI in the Reference list. We also request that you use code versioning and provide a license.

For more information on our code sharing policy and requirements, please see:
https://www.nature.com/nature-research/editorial-policies/reporting-standards#availability-of-computer-code

MATERIALS AVAILABILITY
As a condition of publication in Nature Methods, authors are required to make unique materials promptly available to others without undue qualifications.

Authors reporting new chemical compounds must provide chemical structure, synthesis and characterization details. Authors reporting mutant strains and cell lines are strongly encouraged to use established public repositories.

More details about our materials availability policy can be found at https://www.nature.com/nature-portfolio/editorial-policies/reporting-standards#availability-of-materials

ORCID
Nature Methods is committed to improving transparency in authorship. As part of our efforts in this direction, we are now requesting that all authors identified as ‘corresponding author’ on published papers create and link their Open Researcher and Contributor Identifier (ORCID) with their account on the Manuscript Tracking System (MTS), prior to acceptance. This applies to primary research papers only. ORCID helps the scientific community achieve unambiguous attribution of all scholarly contributions. You can create and link your ORCID from the home page of the MTS by clicking on ‘Modify my Springer Nature account’. For more information please visit please visit www.springernature.com/orcid.

Please do not hesitate to contact me if you have any questions or would like to discuss these revisions further. We look forward to seeing the revised manuscript and thank you for the opportunity to consider your work.

Sincerely,
Rita

Rita Strack, Ph.D.
Senior Editor
Nature Methods

Reviewers' Comments:

Reviewer #1:
Remarks to the Author:

This manuscript describes a very important new multilab study concerning the reliability of smFRET measurements for analyzing distances and dynamics in biomolecules. It is actually a study that probably many FRET researchers have waited for (at least I have) since the authors' previous Nature Methods publication, in which DNA conformation and dynamics were analyzed by multiple labs using smFRET. This new work extends the successful smFRET realiability, precision, and accuracy investigation to proteins and it is truly impressive how well smFRET can analyze also these significantly more complicated biomolecules. The manuscript clearly summarizes the main results and the online methods as well as an extensive Supporting Information file contain all necessary information to follow and possibly reproduce the work from the different labs. I also appreciated that the authors discuss difficulties (for example for the determination of the ⍰ correction factor) in detail and propose possible solutions to overcome the challenges. Overall, this study, which has been performed by internationally renowned labs, presents an important contribution to science and knowledge. It clearly shows that smFRET is an important method for biophysical analysis and is well suited for Nature Methods. I recommend the publication of this manuscript after minor revision (see comments below, which are mainly related to clarity of description).

Abstract: I would change "worldwide" to "international". Asia, Africa, Australia, and South America are not present in the study.

P4: Introduction: Instead of directly describing the sub-field smFRET, I would suggest to shortly introduce FRET in general. I proposed to cite a recent FRET review paper from this very journal (https://doi.org/10.1038/s41592-019-0530-8) and a book chapter that describes the general theory of FRET (https://doi.org/10.1002/9783527656028.ch05).

P4: Was the study of Hellenkamp et al. not performed by 20 labs?

P4: When discussing the differences between DNA and protein analysis, is the controlled bioconjugation (position and orientation within the protein) not also a point that is significantly more challenging when analyzing proteins?

P5: Why was U2AF2 investigated in only 7 labs? There must be a specific reason why 12 labs within a network of collaborating labs did not perform those measurements. Are they too challenging? Is specific equipment and/or analysis required?

P7: Was the dye-labeling also performed by each different lab or were the samples prepared by one lab? Dye labeling is probably an important factor of experimental uncertainly. How was this taken into account. Also, it seems there are 5 labeling positions (all cysteines) and the two dyes (D and A) were stochastically labeled. Does that mean that each dye can take any position? That would also mean that there is a very large distribution of possible D and A fractions and different positions for each system

with a defined fraction of D and A. I assume that this dye distribution will significantly influence the FRET results and it would be interesting if those aspects can be discussed in more detail.

P7: Why only values from 16 labs. What happened to the other 3 labs? I see in the caption of Figure 1 and TableS1 that there were measurement problems. I think that deserves a little more discussion. In the current form, it seems that 3 out of 19 labs face problems, which is more than 15%. Is it likely that the same problems occur for other labs? Are there suggestions to overcome those problems?

P10: If measurements for MalE were performed by 16 labs (or 19), why are the setup dependent parameters and correction factors analyzed for only 8 labs?

P12: Is there any possibility to improve the accuracy and precision of the ⍰ correction factor? For example by using a specific setup, pair of dyes etc.?

Equations: I recommend that the authors double check all their equations. I did not check them all but I found that, for example, Equation S7.1 contains an error: The refractive index should be to the power of 4. I assume it is a typo and the authors used the correct equation for calculating R0.

Reviewer #2:
None

Reviewer #3:
Remarks to the Author:
In their work, Gam et al. present a comparative study to establish error margins for smFRET experiments of proteins. Whereas a previous study (Hellenkamp et al. 2018 Nat Methods, 15, 669) by some of the authors benchmarked smFRET experiments with DNA samples, the current study focuses on proteins that are more heterogeneous both chemically and structurally. The study includes 19 labs and two proteins, MalE (maltose binding protein) and U2AF2 (a spliceosome component). Notably, besides determining lab-to-lab variability of FRET efficiencies and distances, the authors also investigated the reliability of smFRET experiments to identify dynamic properties of the investigated proteins, a task that is clearly new and interesting. For transfer efficiencies, the authors find an uncertainty of ±0.06 across different labs, which is similar to the error found in the previous benchmark study. The authors spotted the sources of this variation between labs in the correction factor determination of the optical system, particularly the gamma-factor, which accounts for the brightness differences and detection differences between donor and acceptor channel. The uncertainty is highest at intermediate FRET efficiencies and using error propagation, the authors present a rigorous analysis of these effects in the Supplementary Information. An analysis of 7 data sets for U2AF2 gave an error of ±0.03 that was reduced to ±0.008 in

an analysis of all data sets (and correction factor determination) by a single person, which demonstrates that instrument variations are not detrimental if correction factors are determined correctly - an important result. In the second part of the work, the authors discuss two methods to identify dynamics in biomolecules: BVA (burst variance analysis) and the combined analysis of donor fluorescence lifetimes and FRET efficiencies. Most labs identified dynamics (or no dynamics) correctly using these methods. The effect of such dynamics on the accuracy of FRET-distance conversions is important. A combined analysis of FRET, anisotropy, and lifetime-FRET plots (Fig. 5) and the use of the positional distribution of the dyes (as given by AV or ACV models) shows that rotational and translational dynamics of the dyes due to their long linkers and dye sticking to protein surfaces can be a source of additional variability. Limits of average limiting anisotropies (<0.2-0.25) to estimate the impact of such processes where confirmed using a diffusion-with-traps (DWT) model and the optimal choice of dyes and labeling sites were stressed. The final part of the paper, a quantitative analysis of U2AF2 using smFRET, lifetime MEM analysis, filtered FCS, NMR and SAXS seems to be less informative, except maybe for researcher working on this particular protein.
Overall, this joint effort provides an excellent overview over the potential and limits of smFRET experiments together with a rich set of analysis tools that allow researcher to improve their analysis (if they are not already doing it anyway). The study will particularly be helpful for future integrative approaches in which different methods (smFRET, SAXS, SANS, NMR, EPR) are combined to generate structural ensembles of biomolecules. I am therefore very positive, but I would like to ask the authors to address a few comments:

1. Stressing the number of 19 labs in the abstract and discussion seems a bit exaggerated as only FRET of the protein MalE has been studied by 19 labs, whereas U2AF2 was studied by 7 labs, and also dynamics where studied by 7-8 labs. This is still a large number and I don’t think this study requires any exaggeration.

2. The determination of gamma values and other correction factors has been done in a global manner, i.e., including experiments of different variants or conditions. This was necessary as FRET distributions of MalE or U2AF2 are unimodal. Yet, a significant difference in E (less in S) between two populations is required to determine the gamma factor precisely. It would have been interesting to determine the uncertainty in gamma as function of the separation of FRET peaks. For two FRET-peaks with coordinates (E, S) and (E+ΔE, S+ΔS) in the E-S plot that are corrected for background, leakage, and acceptor direct excitation, and assuming identical localization error dS and dE (which should be proportional to $1/\sqrt{N}$ with N being the average photon number), the error in determining gamma should scale as $1/\Delta E$, which seems significant to me. It would be helpful to see a more rigorous analysis in the paper.

3. Related to point 2, gamma determinations with dye solutions are an alternative used by some labs. Clearly, as a downside, dyes are not in a native environment, i.e., close to the protein surface, yet, shot

noise doesn't play a role in correction factor determinations. The authors might want to comment on the pros and cons of the two strategies.

4. I didn't find the numbers used for the factors l1 and l2 for anisotropy measurements in a confocal setup (Supplement p. 21). I am asking since determining these factors is often associated with a significant error (personal experience). Did the authors determine the values for their systems or did they take literature values?

5. It was not clear to me how the authors chose the correct averaging regime for FRET-calculations based on structural models given that the dyes do not only rotate but also translationally diffuse within the AV (AVC) volume. When analyzing the effect of donor-quenching (Supplementary Note 10), the authors used Brownian diffusion of the dye in the AV-volume. For distance calculations in absence of quenching, the authors computed FRET efficiency averaged model distances between the two dyes (p. 15, 2nd paragraph) and ref. 4 indicates that a static average across positions in the AV volume was chosen. Since translational motion across significant distances happens at a timescale of ns as suggested by the dye-diffusion coefficient provided by the authors (10 Å2/ns for one dye), I was wondering whether the authors take the partial averaging in the AV-volume during the dye fluorescence lifetime into account, e.g., via Brownian dynamics simulations alike those used to analyse donor quenching?

6. Related to point 5, recent results (Klose et al. 2021 Biophys J, 120, 4842) suggest that rotamer libraries seem superior to coarse-grained AV calculations for computing FRET values from structures. Please comment.

Author Rebuttal to Initial comments

Response to the Reviewers:

Reviewer #1:
Remarks to the Author:
This manuscript describes a very important new multilab study concerning the reliability of smFRET measurements for analyzing distances and dynamics in biomolecules. It is actually a study that probably many FRET researchers have waited for (at least I have) since the authors' previous Nature Methods publication, in which DNA conformation and dynamics were analyzed by multiple labs using smFRET. This new work extends the successful smFRET realiability, precision, and accuracy investigation to proteins and it is truly impressive how well smFRET can analyze also these significantly more complicated biomolecules. The manuscript clearly summarizes the main results and the online methods as well as an extensive Supporting Information file contain all necessary information to follow and possibly reproduce the work from the different labs. I also appreciated that the authors discuss difficulties (for example for the determination of the γ correction factor) in detail and propose possible solutions to overcome the challenges. Overall, this study, which has been performed by internationally renowned labs, presents an important contribution to science and knowledge. It clearly shows that smFRET is an important method for biophysical analysis and is well suited for Nature Methods. I recommend the publication of this manuscript after minor revision (see comments below, which are mainly related to clarity of description).

We thank the reviewer for her/his support for this study.

Abstract: I would change "worldwide" to "international". Asia, Africa, Australia, and South America are not present in the study.

Agreed. We have changed the wording as requested.

P4: Introduction: Instead of directly describing the sub-field smFRET, I would suggest to shortly introduce FRET in general. I proposed to cite a recent FRET review paper from this very journal (https://doi.org/10.1038/s41592-019-0530-8) and a book chapter that describes the general theory of FRET (https://doi.org/10.1002/9783527656028.ch05).

We have edited the introduction as recommended by the reviewer.

P4: Was the study of Hellenkamp et al. not performed by 20 labs?

According to Thorsten Hugel, there were 21 labs in total with 20 labs in the study being "blind" with respect to the study. We have corrected this in the text.

P4: When discussing the differences between DNA and protein analysis, is the controlled bioconjugation (position and orientation within the protein) not also a point that is significantly more challenging when analyzing proteins?

We have added a sentence regarding the labeling position and orientation challenges within the protein. **"Site-specific labeling of proteins usually requires the introduction of point mutations (typically cysteines), which can pose further complications with respect to the labeling procedures depending on the structure and nature of the protein.[1]"**

P5: Why was U2AF2 investigated in only 7 labs? There must be a specific reason why 12 labs within a network of collaborating labs did not perform those measurements. Are they too challenging? Is specific equipment and/or analysis required?

We sent an email to all 19 participating laboratories inviting them to perform measurements with the second protein. Eight labs agreed to measure the second protein. Due to a mis-communication, data from one of these laboratories was misinterpreted as corrected FRET data, when it had not been corrected. By the time this miscommunication was noticed, it was too late to correct the data from this laboratory and keep the study as truly blind, leaving us with 7 laboratories involved in the U2AF2 measurements.

P7: Was the dye-labeling also performed by each different lab or were the samples prepared by one lab? Dye labeling is probably an important factor of experimental uncertainly. How was this taken into account. Also, it seems there are 5 labeling positions (all cysteines) and the two dyes (D and A) were stochastically labeled. Does that mean that each dye can take any position? That would also mean that there is a very large distribution of possible D and A fractions and different positions for each system with a defined fraction of D and A. I assume that this dye distribution will significantly influence the FRET results and it would be interesting if those aspects can be discussed in more detail.

To make the study as blind as possible, the participating groups were not informed what protein they were dealing with. If groups were expected to label the protein, it would then be advisable to test the functionality of the protein, which is difficult to do if you do not know which protein it is. Hence, the protein labeling was performed in the Cordes lab for MalE and in the Lamb lab for U2AF2. The labeled protein was then sent to the different laboratories. We have now clarified this point in the results. **"To avoid additional complexity and to restrict any pre-knowledge regarding the samples, the proteins were labeled and checked for functionality before being delivered to the participants."**
The labeling and functionality of the protein are additional challenges that we did not investigate in this study to avoid additional complexity, e.g., due to a loss of functionality during the labeling procedure. While we did not ask the different labs to label the proteins individually, we note that the accuracy of smFRET experiments is largely independent of the labeling efficiency as long as the proteins survive the labeling procedure and a sufficient amount of double-labeled molecules is detected.

Regarding the labeling positions, we apologize for the confusion. Each of the individual MalE mutants contained only a single cysteine pair. We have clarified this better in the text in the caption of Fig. 1 by adding the statement "**Note, each mutant only contains one cysteine pair and was measured using the Alexa546-Alexa647 FRET pair.**" and the results section.

P7: Why only values from 16 labs. What happened to the other 3 labs? I see in the caption of Figure 1 and TableS1 that there were measurement problems. I think that deserves a little more discussion. In the current form, it seems that 3 out of 19 labs face problems, which is more than 15%. Is it likely that the same problems occur for other labs? Are there suggestions to overcome those problems?

We now mention some of the difficulties the three labs encountered in the study of the first protein system in the main text and in Supplementary Note 2.

P10: If measurements for MalE were performed by 16 labs (or 19), why are the setup dependent parameters and correction factors analyzed for only 8 labs?

During the course of the more detailed analysis of U2AF2, we collected the detailed information from the eight laboratories involved (see our response above regarding labs that measured U2AF2). We believe that they are sufficient to get an idea of the distribution of values within the community.

P12: Is there any possibility to improve the accuracy and precision of the γ correction factor? For example by using a specific setup, pair of dyes etc.?

We have considered what would be the optimal way of determining the detection correction factor. The best approach we are currently aware of depends on the properties of the sample(s) and the type of data that is collected. An evaluation of intensity and lifetime based approaches for gamma factor determination is currently ongoing (Manuscript in preparation; Popara et al). As requested also by reviewer 3, point 3, we now discuss the approach of using dye standards for determining the gamma factor and the pros and cons of this and other approaches in Supplementary Note 2. Theoretically, the uncertainty of the gamma factor also scales with the inverse of the FRET efficiency contrast (see answer to reviewer #3, point 2 below) although, for FRET contrasts above 0.2, this does not appear to play a role experimentally (Vandenberk et al, J Phys ChemB, 2018).

Equations: I recommend that the authors double check all their equations. I did not check them all but I found that, for example, Equation S7.1 contains an error: The refractive index should be to the power of 4. I assume it is a typo and the authors used the correct equation for calculating R0.

We thank the reviewer for catching this typo. We did use the correct formula for the calculation of R_0. We have double-checked all equations in the paper.

Reviewer #2:
None

Reviewer #3:

Remarks to the Author:

In their work, Gam et al. present a comparative study to establish error margins for smFRET experiments of proteins. Whereas a previous study (Hellenkamp et al. 2018 Nat Methods, 15, 669) by some of the authors benchmarked smFRET experiments with DNA samples, the current study focuses on proteins that are more heterogeneous both chemically and structurally. The study includes 19 labs and two proteins, MalE (maltose binding protein) and U2AF2 (a spliceosome component). Notably, besides determining lab-to-lab variability of FRET efficiencies and distances, the authors also investigated the reliability of smFRET experiments to identify dynamic properties of the investigated proteins, a task that is clearly new and interesting. For transfer efficiencies, the authors find an uncertainty of ±0.06 across different labs, which is similar to the error found in the previous benchmark study. The authors spotted the sources of this variation between labs in the correction factor determination of the optical system, particularly the gamma-factor, which accounts for the brightness differences and detection differences between donor and acceptor channel. The uncertainty is highest at intermediate FRET efficiencies and using error propagation, the authors present a rigorous analysis of these effects in the Supplementary Information. An analysis of 7 data sets for U2AF2 gave an error of ±0.03 that was reduced to ±0.008 in an analysis of all data sets (and correction factor determination) by a single person, which demonstrates that instrument variations are not detrimental if correction factors are determined correctly - an important result. In the second part of the work, the authors discuss two methods to identify dynamics in biomolecules: BVA (burst variance analysis) and the combined analysis of donor fluorescence lifetimes and FRET efficiencies. Most labs identified dynamics (or no dynamics) correctly using these methods. The effect of such dynamics on the accuracy of FRET-distance conversions is important. A combined analysis of FRET, anisotropy, and lifetime-FRET plots (Fig. 5) and the use of the positional distribution of the dyes (as given by AV or ACV models) shows that rotational and translational dynamics of the dyes due to their long linkers and dye sticking to protein surfaces can be a source of additional variability. Limits of average limiting anisotropies (<0.2-0.25) to estimate the impact of such processes where confirmed using a diffusion-with-traps (DWT) model and the optimal choice of dyes and labeling sites were stressed. The final part of the paper, a quantitative analysis of U2AF2 using smFRET, lifetime MEM analysis, filtered FCS, NMR and SAXS seems to be less informative, except maybe for researcher working on this particular protein. Overall, this joint effort provides an excellent overview over the potential and limits of smFRET experiments together with a rich set of analysis tools that allow researcher to improve their analysis (if they are not already doing it anyway). The study will particularly be helpful for future integrative approaches in which different methods (smFRET, SAXS, SANS, NMR, EPR) are combined to generate structural ensembles of biomolecules. I am therefore very positive, but I would like to ask the authors to address a few comments:

We thank the reviewer for her/his detailed reading of the paper and support of the presented work.

1. Stressing the number of 19 labs in the abstract and discussion seems a bit exaggerated as only

FRET of the protein MalE has been studied by 19 labs, whereas U2AF2 was studied by 7 labs, and also dynamics where studied by 7-8 labs. This is still a large number and I don't think this study requires any exaggeration.

We have toned down the wording in the manuscript and give the number of laboratories only where necessary.

2. The determination of gamma values and other correction factors has been done in a global manner, i.e., including experiments of different variants or conditions. This was necessary as FRET distributions of MalE or U2AF2 are unimodal. Yet, a significant difference in E (less in S) between two populations is required to determine the gamma factor precisely. It would have been interesting to determine the uncertainty in gamma as function of the separation of FRET peaks. For two FRET-peaks with coordinates (E, S) and (E+ΔE, S+ΔS) in the E-S plot that are corrected for background, leakage, and acceptor direct excitation, and assuming identical localization error dS and dE (which should be proportional to 1/√N with N being the average photon number), the error in determining gamma should scale as 1/ΔE, which seems significant to me. It would be helpful to see a more rigorous analysis in the paper.

This is an interesting point. We have now looked from a theoretical side into how the error in gamma depends on the accuracy of E and S when two populations are present. This analysis confirms that the error scales approximately as 1/ΔE, as correctly predicted by the reviewer. We have added this information to the SI in Supplementary Note 2.

From the experimental side, the effect of the FRET efficiency contrast on the gamma determination has previously been investigated for multiple dye pairs by Vandenberk et al. (2018), Figure S4, where they found no clear dependence between the gamma factor and ΔE across a wide range of values.

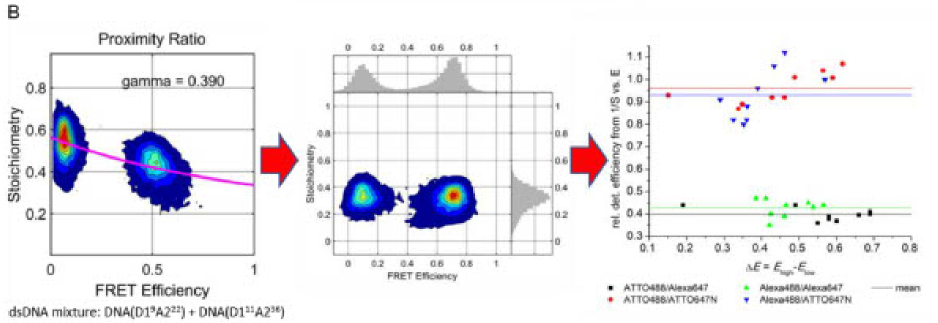

Figure S4 from Vandenberk, N., Barth, A., Borrenberghs, D., Hofkens, J., & Hendrix, J. (2018). Evaluation of Blue and Far-Red Dye Pairs in Single-Molecule Förster Resonance Energy Transfer Experiments. *J Phys Chem B*, *122*(15), 4249-4266. https://doi.org/10.1021/acs.jpcb.8b00108

3. Related to point 2, gamma determinations with dye solutions are an alternative used by some

labs. Clearly, as a downside, dyes are not in a native environment, i.e., close to the protein surface, yet, shot noise doesn't play a role in correction factor determinations. The authors might want to comment on the pros and cons of the two strategies.

This point is similar to one of the points raised by reviewer 1. In our discussion regarding the determination of the gamma factor (Supplementary Note 2), we have now discussed the approach of using dye standards for determining the gamma factor and discuss the pros and cons of this and other approaches. Changes in the quantum yield and spectral shifts that occur in the sample of interest will not be corrected for via these approaches. When there is no spectral shift, or the shift is minimal, these approaches work well for determining crosstalk and direction excitation. We discuss this now in more detail in Supplementary Note 2.

4. I didn't find the numbers used for the factors l1 and l2 for anisotropy measurements in a confocal setup (Supplement p. 21). I am asking since determining these factors is often associated with a significant error (personal experience). Did the authors determine the values for their systems or did they take literature values?

We have added information regarding the correction factors l1 and l2 in the Supplementary Note 8, after equation 8.4.

"Since correction for polarization mixing is only needed when using a high NA objective, they were not needed for the analysis of ensemble TCSPC measurements of single-cysteine mutants (Supplementary Table 11) and the l1 and l2 correction factors were set to zero. For the anisotropy analysis of sub-ensemble data (Supplementary Table 5) acquired using confocal single-molecule setups, values of l1 = 0.0308 and l2 = 0.0368 were used for samples labeled with Alexa Fluor 546 or Atto532 as the donor dye, and l1 = 0.0175 and l2 = 0.0526 for samples labeled with Alexa Fluor 488 as the donor dye. These values were determined with the following procedure using the enhanced Yellow Fluorescent Protein (eYFP). eYFP has a slow rotational correlation time of ρ = 16 ns, a monoexponential lifetime decay with τ = 3.2 ns and a fundamental anisotropy of r0 = 0.39. To determine the l1 and l2 correction factors, we performed a global fit of polarization resolved signals of eYFP with fixed ρ and r0. When τ is previously determined from a measurement at the magic angle, it can be fixed as well in the fit, reducing the number of free fit parameters.[21]"

Furthermore, we also mention this in Supplementary Note 18, Lab #2, where we added the following sentences:

"Setup #1 for samples labeled with Alexa Fluor 546 – Alexa Fluor 647, Alexa Fluor 546 – AbberiorSTAR635P and Atto 532 – Atto 643: ... To account for polarization mixing in the microscope objective, eYFP (enhanced Yellow Fluorescent Protein) was used as a calibration sample using the procedure described in Supplementary Note 8. The polarization correction factors were determined to be: l1 = 0.0308 and l2 = 0.0368.

Setup #2 for samples labeled with Alexa Fluor 488 – Alexa Fluor 647: ... As for setup #1, the correction factors for polarization mixing in the microscope objective were determined to be l1 = 0.0175 and l2 = 0.0526."

5. It was not clear to me how the authors chose the correct averaging regime for FRET-calculations based on structural models given that the dyes do not only rotate but also translationally diffuse within the AV (AVC) volume. When analyzing the effect of donor-quenching (Supplementary Note 10), the authors used Brownian diffusion of the dye in the AV-volume. For distance calculations in absence of quenching, the authors computed FRET efficiency averaged model distances between the two dyes (p. 15, 2nd paragraph) and ref. 4 indicates that a static average across positions in the AV volume was chosen. Since translational motion across significant distances happens at a timescale of ns as suggested by the dye-diffusion coefficient provided by the authors (10 Å2/ns for one dye), I was wondering whether the authors take the partial averaging in the AV-volume during the dye fluorescence lifetime into account, e.g., via Brownian dynamics simulations alike those used to analyse donor quenching?

In a detailed study, we have found that fast rotational averaging and slow translational diffusion with respect to the fluorescence lifetime is a suitable approximation, which is also the typical assumption used in the field. We now write this explicitly in the main text on page 15. As there is no easy analytical model to describe the diffusional behavior of a dye coupled to a biomolecule, the Seidel lab performed all-atom molecular dynamics simulations of dyes coupled to rigid RNA rulers (SI reference 32, which is in preparation) and coarse grained simulations (Peulen, T. O., Opanasyuk, O., & Seidel, C. A. M. (2017). Combining Graphical and Analytical Methods with Molecular Simulations To Analyze Time-Resolved FRET Measurements of Labeled Macromolecules Accurately. *J Phys Chem B*, *121*(35), 8211-8241. https://doi.org/10.1021/acs.jpcb.7b03441) to investigate their behavior. We compared the simulations with experimental high-precision FRET measurements of the same constructs according to Wozniak et al. (Wozniak, A. K., Schröder, G. F., Grubmüller, H., Seidel, C. A., & Oesterhelt, F. (2008). Single-molecule FRET measures bends and kinks in DNA. *Proc Natl Acad Sci U S A*, *105*(47), 18337-18342. https://doi.org/10.1073/pnas.0800977105). The comparison for a set of more than 20 RNA rulers with distinct DA-distances showed the approximation of fast rotational averaging, i.e. $\langle\kappa^2\rangle=2/3$, and slow positional averaging with respect to the fluorescence lifetime provides the best description of the experimental data. We can rationalize this result by taking into account that the errors in both approximations cancel out: Slower rotational averaging results in apparent longer average distances (see Fig. 4 in Hummer, G. & Szabo, A. Dynamics of the Orientational Factor in Fluorescence Resonance Energy Transfer. J. Phys. Chem. B 121, 3331-3339 (2017)), while fast positional averaging during the donor excited state lifetime results in smaller average distances. Thus, the errors of both assumptions cancel out, to a large extent. We also found that this approximation is the most reasonable for biomolecular studies. Notably, the experimental and theoretical diffusion coefficients for translational and rotational diffusion agreed well in our studies.

6. Related to point 5, recent results (Klose et al. 2021 Biophys J, 120, 4842) suggest that rotamer

libraries seem superior to coarse-grained AV calculations for computing FRET values from structures. Please comment.

Certainly, AV calculations is only one of several available approaches for converting the measured FRET efficiency values into interdye distances. The use of rotamer libraries does provide a more accurate description of the fluorophore and its linker. However, neither rotamer libraries nor AV calculations include the sticking of the dyes to the surface of biomolecules. Thus, we have applied the ACV approach here, which is calibrated using the experimental data (i.e., residual anisotropies).
On page 15, we have now added a statement that there are other approaches for estimating the position and averaging of the fluorophores, which are attached to the biomolecule of interest.

In addition to the issues raised by the reviewers, for clarity we have also added a table at the beginning of Supplementary Note 18 summarizing the important parameters of the different setups used in this study.

Decision Letter, first revision:

Dear Don,

Thank you for submitting your revised manuscript "Reliability and accuracy of single-molecule FRET studies for characterization of structural dynamics and distances in proteins" (NMETH-AS50048A). It has now been seen by the original referees and their comments are below. The reviewers find that the paper has improved in revision, and therefore we'll be happy in principle to publish it in Nature Methods, pending minor revisions to comply with our editorial and formatting guidelines.

We are now performing detailed checks on your paper and will send you a checklist detailing our editorial and formatting requirements in about a week. Please do not upload the final materials and make any revisions until you receive this additional information from us.

TRANSPARENT PEER REVIEW
Nature Methods offers a transparent peer review option for new original research manuscripts submitted from 17th February 2021. We encourage increased transparency in peer review by publishing the reviewer comments, author rebuttal letters and editorial decision letters if the authors agree. Such peer review material is made available as a supplementary peer review file. Please state in the cover letter 'I wish to participate in transparent peer review' if you want to opt in, or 'I do not wish to participate in transparent peer review' if you don't. Failure to state your preference will result in delays in accepting your manuscript for publication.

Please note: we allow redactions to authors' rebuttal and reviewer comments in the interest of confidentiality. If you are concerned about the release of confidential data, please let us know specifically what information you would like to have removed. Please note that we cannot incorporate redactions for any other reasons. Reviewer names will be published in the peer review files if the reviewer signed the comments to authors, or if reviewers explicitly agree to release their name. For more information, please refer to our FAQ page.

ORCID
IMPORTANT: Non-corresponding authors do not have to link their ORCIDs but are encouraged to do so. Please note that it will not be possible to add/modify ORCIDs at proof. Thus, please let your co-authors know that if they wish to have their ORCID added to the paper they must follow the procedure described in the following link prior to acceptance:
https://www.springernature.com/gp/researchers/orcid/orcid-for-nature-research

Thank you again for your interest in Nature Methods. Please do not hesitate to contact me if you have any questions. We will be in touch again soon.

Sincerely,
Rita

Rita Strack, Ph.D.
Senior Editor
Nature Methods

Reviewer #1 (Remarks to the Author):

The authors have carefully revised their manuscript and appropriately answered to the comments of both reviewers. The additional explanations and data have further improved the clarity and quality of the manuscript.
I recommend the publication of this revised version in Nature Methods. No more modifications necessary.

Reviewer #3 (Remarks to the Author):

I thank the authors for providing detailed answers to my points.

Final Decision Letter:

Dear Don,

I am pleased to inform you that your Analysis, "Reliability and accuracy of single-molecule FRET studies for characterization of structural dynamics and distances in proteins", has now been accepted for publication in Nature Methods. Your paper is tentatively scheduled for publication in our May print issue, and will be published online prior to that. The received and accepted dates will be August 2, 2022 and Jan 31, 2023. This note is intended to let you know what to expect from us over the next month or so, and to let you know where to address any further questions.

Acceptance is conditional on the data in the manuscript not being published elsewhere, or announced in the print or electronic media, until the embargo/publication date. These restrictions are not intended to

deter you from presenting your data at academic meetings and conferences, but any enquiries from the media about papers not yet scheduled for publication should be referred to us.

Over the next few weeks, your paper will be copyedited to ensure that it conforms to Nature Methods style. Once your paper is typeset, you will receive an email with a link to choose the appropriate publishing options for your paper and our Author Services team will be in touch regarding any additional information that may be required.

After the grant of rights is completed, you will receive a link to your electronic proof via email with a request to make any corrections within 48 hours. If, when you receive your proof, you cannot meet this deadline, please inform us at rjsproduction@springernature.com immediately.

Please note that <i>Nature Methods</i> is a Transformative Journal (TJ). Authors may publish their research with us through the traditional subscription access route or make their paper immediately open access through payment of an article-processing charge (APC). Authors will not be required to make a final decision about access to their article until it has been accepted. Find out more about Transformative Journals

Authors may need to take specific actions to achieve compliance with funder and institutional open access mandates. If your research is supported by a funder that requires immediate open access (e.g. according to Plan S principles) then you should select the gold OA route, and we will direct you to the compliant route where possible. For authors selecting the subscription publication route, the journal's standard licensing terms will need to be accepted, including self-archiving policies. Those licensing terms will supersede any other terms that the author or any third party may assert apply to any version of the manuscript.

You will not receive your proofs until the publishing agreement has been received through our system.

If you have any questions about our publishing options, costs, Open Access requirements, or our legal forms, please contact ASJournals@springernature.com

Your paper will now be copyedited to ensure that it conforms to Nature Methods style. Once proofs are generated, they will be sent to you electronically and you will be asked to send a corrected version within 24 hours. It is extremely important that you let us know now whether you will be difficult to contact over the next month. If this is the case, we ask that you send us the contact information (email,

phone and fax) of someone who will be able to check the proofs and deal with any last-minute problems.

If, when you receive your proof, you cannot meet the deadline, please inform us at rjsproduction@springernature.com immediately.

Once your manuscript is typeset and you have completed the appropriate grant of rights, you will receive a link to your electronic proof via email with a request to make any corrections within 48 hours. If, when you receive your proof, you cannot meet this deadline, please inform us at rjsproduction@springernature.com immediately.

Once your paper has been scheduled for online publication, the Nature press office will be in touch to confirm the details.

If you have posted a preprint on any preprint server, please ensure that the preprint details are updated with a publication reference, including the DOI and a URL to the published version of the article on the journal website.

Once your paper has been scheduled for online publication, the Nature press office will be in touch to confirm the details.

Content is published online weekly on Mondays and Thursdays, and the embargo is set at 16:00 London time (GMT)/11:00 am US Eastern time (EST) on the day of publication. If you need to know the exact publication date or when the news embargo will be lifted, please contact our press office after you have submitted your proof corrections. Now is the time to inform your Public Relations or Press Office about your paper, as they might be interested in promoting its publication. This will allow them time to prepare an accurate and satisfactory press release. Include your manuscript tracking number NMETH-AS50048B and the name of the journal, which they will need when they contact our office.

About one week before your paper is published online, we shall be distributing a press release to news organizations worldwide, which may include details of your work. We are happy for your institution or funding agency to prepare its own press release, but it must mention the embargo date and Nature Methods. Our Press Office will contact you closer to the time of publication, but if you or your Press Office have any inquiries in the meantime, please contact press@nature.com.

To assist our authors in disseminating their research to the broader community, our SharedIt initiative provides you with a unique shareable link that will allow anyone (with or without a subscription) to read the published article. Recipients of the link with a subscription will also be able to download and print the PDF.

As soon as your article is published, you will receive an automated email with your shareable link.

You can now use a single sign-on for all your accounts, view the status of all your manuscript submissions and reviews, access usage statistics for your published articles and download a record of your refereeing activity for the Nature journals.

Nature Portfolio journals encourage authors to share their step-by-step experimental protocols on a protocol sharing platform of their choice. Nature Portfolio 's Protocol Exchange is a free-to-use and open resource for protocols; protocols deposited in Protocol Exchange are citable and can be linked from the published article. More details can found at www.nature.com/protocolexchange/about.

Please note that you and any of your coauthors will be able to order reprints and single copies of the issue containing your article through Nature Portfolio's reprint website, which is located at http://www.nature.com/reprints/author-reprints.html. If there are any questions about reprints please send an email to author-reprints@nature.com and someone will assist you.

Please feel free to contact me if you have questions about any of these points.

Best regards,
Rita

Rita Strack, Ph.D.
Senior Editor
Nature Methods
